# Distribution patterns of tau pathology in progressive supranuclear palsy

**DOI:** 10.1007/s00401-020-02158-2

**Published:** 2020-05-07

**Authors:** Gabor G. Kovacs, Milica Jecmenica Lukic, David J. Irwin, Thomas Arzberger, Gesine Respondek, Edward B. Lee, David Coughlin, Armin Giese, Murray Grossman, Carolin Kurz, Corey T. McMillan, Ellen Gelpi, Yaroslau Compta, John C. van Swieten, Laura Donker Laat, Claire Troakes, Safa Al-Sarraj, John L. Robinson, Sigrun Roeber, Sharon X. Xie, Virginia M.- Y. Lee, John Q. Trojanowski, Günter U. Höglinger

**Affiliations:** 1 Center for Neurodegenerative Disease Research (CNDR), Institute On Aging and Department of Pathology & Laboratory Medicine, University of Pennsylvania, 3600 Spruce Street, 3 Maloney Building, Philadelphia, PA 19104-4283 USA; 2 Tanz Centre for Research in Neurodegenerative Disease (CRND) and Department of Laboratory Medicine and Pathobiology, University of Toronto, 60 Leonard Ave, Krembil Discovery Tower, Toronto, ON M5T 0S8 Canada; 3 Laboratory Medicine Program and Krembil Brain Institute, University Health Network, Toronto, ON Canada; 4 German Center for Neurodegenerative Diseases (DZNE), Munich, Germany; 5 Clinic of Neurology, CCS, University of Belgrade, Belgrade, Republic of Serbia; 6 Department of Neurology, University of Pennsylvania, Philadelphia, USA; 7 Department of Psychiatry and Psychotherapy, University Hospital, LMU Munich, Munich, Germany; 8 Center for Neuropathology and Prion Research, LMU Munich, Munich, Germany; 9 Munich Cluster for Systems Neurology (SyNergy), Munich, Germany; 10 Department of Neurology, Klinikum Rechts der Isar, Technical University of Munich, Munich, Germany; 11 Frontotemporal Degeneration Center, University of Pennsylvania, Philadelphia, USA; 12 Neurological Tissue Bank and Neurology Department, Hospital Clínic de Barcelona, Universitat de Barcelona, IDIBAPS, CERCA, Barcelona, Catalonia Spain; 13 Parkinson’s Disease & Movement Disorders Unit, Hospital Clínic / IDIBAPS / CIBERNED (CB06/05/0018-ISCIII) / European Reference Network for Rare Neurological Diseases (ERN-RND) / Institut de Neurociències (Maria de Maeztu Center), Universitat de Barcelona, Barcelona, Catalonia Spain; 14 Department of Neurology, Erasmus Medical Centre, Rotterdam, The Netherlands; 15 Department Clinical Genetics, Erasmus Medical Center, Rotterdam, The Netherlands; 16 London Neurodegenerative Diseases Brain Bank, Institute of Psychiatry, Psychology and Neuroscience, Kings College London, London, UK; 17 Department of Biostatistics, Epidemiology and Informatics, University of Pennsylvania, Philadelphia, USA; 18 Department of Neurology, Hannover Medical School, Carl-Neuberg-Str. 1, 30625 Hannover, Germany; 19 Present Address: Department of Neurosciences, University of California, La Jolla, San Diego, CA USA; 20 Present Address: Institute of Neurology, Medical University of Vienna, Vienna, Austria

**Keywords:** Coiled body, Neurofibrillary tangle, Progressive supranuclear palsy, Propagation, Richardson syndrome, Sequential involvement, Stage, Tau, Tauopathy, Tufted astrocyte

## Abstract

Progressive supranuclear palsy (PSP) is a 4R-tauopathy predominated by subcortical pathology in neurons, astrocytes, and oligodendroglia associated with various clinical phenotypes. In the present international study, we addressed the question of whether or not sequential distribution patterns can be recognized for PSP pathology. We evaluated heat maps and distribution patterns of neuronal, astroglial, and oligodendroglial tau pathologies and their combinations in different clinical subtypes of PSP in postmortem brains. We used conditional probability and logistic regression to model the sequential distribution of tau pathologies across different brain regions. Tau pathology uniformly predominates in the neurons of the pallido-nigro-luysian axis in different clinical subtypes. However, clinical subtypes are distinguished not only by total tau load but rather cell-type (neuronal versus glial) specific vulnerability patterns of brain regions suggesting distinct dynamics or circuit-specific segregation of propagation of tau pathologies. For Richardson syndrome (*n* = 81) we recognize six sequential steps of involvement of brain regions by the combination of cellular tau pathologies. This is translated to six stages for the practical neuropathological diagnosis by the evaluation of the subthalamic nucleus, globus pallidus, striatum, cerebellum with dentate nucleus, and frontal and occipital cortices. This system can be applied to further clinical subtypes by emphasizing whether they show caudal (cerebellum/dentate nucleus) or rostral (cortical) predominant, or both types of pattern. Defining cell-specific stages of tau pathology helps to identify preclinical or early-stage cases for the better understanding of early pathogenic events, has implications for understanding the clinical subtype-specific dynamics of disease-propagation, and informs tau-neuroimaging on distribution patterns.

**Electronic supplementary material:**

The online version of this article (10.1007/s00401-020-02158-2) contains supplementary material, which is available to authorized users.

## Introduction

Progressive supranuclear palsy (PSP) is a four-repeat (4R) tauopathy that belongs to the group of frontotemporal lobar degeneration (FTLD-tau) disorders [34]. The neuropathological diagnosis of PSP is based on the presence of neurofibrillary tangles and threads in subcortical nuclei together with the presence of tufted astrocytes [5, 13]. In addition, oligodendroglial coiled bodies and diffuse cytoplasmic immunoreactivity in neurons can be observed as well [9, 18]. Following the proposal of typical, atypical, and combined cases of PSP by Lantos [26], Williams et al. provided evidence for biochemical and tau pathology load differences between PSP with Parkinsonism (PSP-P) and the classical clinical phenotype Richardson syndrome (PSP-RS) [47, 48]. Soon thereafter, further clinical phenotypes with PSP-type tau pathology were described [6]. In 2017, the Movement Disorders Society suggested clinical diagnostic criteria to recognize these distinct clinical subtypes as PSP-RS, PSP-P, PSP with corticobasal syndrome (PSP-CBS), PSP with progressive gait freezing (PSP-PGF), PSP with predominant ocular motor dysfunction (PSP-OM), with predominant postural instability (PSP-PI), with predominant frontal presentation (PSP-F), or with predominant speech and language disorder (PSP-SL) [14].

Neuropathological studies on smaller cohorts suggest differences in the burden of tau pathology between clinical subtypes. Williams et al. noted in 2007 that the mean severity of pathology in all regions of the PSP-RS group (*n* = 22) was higher than in PSP-P (*n* = 14) and PSP with pure akinesia with gait freezing (currently called PSP-PGF, *n* = 6), and the overall tau load was significantly higher in PSP-RS than in PSP-P. Sakae et al. compared PSP-RS (*n* = 31) cases with PSP-F (*n* = 15) and found increased tau burden only in the superior frontal gyrus gray matter and inferior temporal gyrus white matter in PSP-F [36]. Tsuboi et al. examined cases (*n* = 5) presenting with PSP-CBS and concluded that this is most likely due to either concurrent cortical pathology, or to the primary pathology of PSP affecting cortical areas that are primarily and commonly affected by corticobasal degeneration (CBD), another 4R tauopathy [45]. Ling et al. also examined PSP-CBS cases (*n* = 10) and demonstrated that the overall severity of tau pathology was the same between PSP-CBS and PSP-RS but with a shift of tau burden towards the cortical regions [27]. On the other hand, the rare PSP-PGF variant showed almost no cortical tau pathology, but severe degeneration of the globus pallidus, substantia nigra, and subthalamic nucleus, hence called also pallido-nigro-luysian degeneration [1].

In addition to the recognition of clinical subtypes, a novel concept raises the possibility of propagation of pathological tau in PSP as well as other tauopathies [11] providing a potential therapeutic target [16, 34]. Indeed, sequential distribution patterns have been recognized for tau pathologies such as neurofibrillary degeneration in Alzheimer’s disease (AD) [3], Pick’s disease [15], argyrophilic grain disease [35], or astrocytic tau pathologies [24] as well as for other proteinopathies such as beta-amyloid, alpha-synuclein or TDP-43 (for review see: [19]). Regarding PSP, Williams et al. proposed a scoring system of tau pathology allowing the recognition of sequential distribution patterns in PSP-RS [48]. However, due to the variability of tau cytopathologies and clinical phenotypes, a staging system such as that developed focusing only on neuronal protein depositions exemplified by neurofibrillary tangles [3] or Lewy bodies [4] has not yet been proposed for PSP. Based on these unresolved issues, we set up an international group to collect different clinical subtypes with PSP pathology. We compared the distribution patterns of tau cytopathologies and used heat maps and conditional probability matrix to evaluate whether or not sequential patterns of tau pathology can be recognized and whether the clinical subtypes show distinct distribution patterns of tau cytopathologies.

## Materials and methods

### Case cohort

This study includes 206 individuals in banked collection of brains from longitudinally followed subjects at the Center of Neurodegenerative Disease Research (CNDR) Brain Bank at the University of Pennsylvania, Philadelphia, PA and brain banks in Munich, Germany, Barcelona, Spain, London, UK, and Rotterdam, The Netherlands (Table 1). Cases for the study were selected based on the presence of globose NFTs in the subthalamic nucleus, substantia nigra, and globus pallidus together with tufted astrocytes in the striatum and eventually in the frontal cortex. Cases were grouped following PSP-RS, PSP-P, PSP-CBS, PSP-PGF, PSP-OM, PSP-PI, PSP-F, or PSP-SL using the operationalized Movement Disorders Society diagnostic PSP criteria [14], by movement disorders experts (DI, GR) based on the records in the patient’s *ante mortem* clinical files, as described previously [31]. Data were obtained from clinical charts and may be incomplete and underestimated. In order to minimalize this retrospective limitation, we relied on clinically obvious signs and symptoms relevant to our analysis, which were commonly reported by patient, caregiver and/or documented by doctor. Clinical features were considered present if specifically mentioned in the clinical notes. They were considered absent if they were specifically mentioned as absent, or if they were not mentioned.

**Table 1:** Demographic data of cases examined in this study

PSP syndrome		Country		Sex		Total	Mean Age	SE	Mean duration (*n*)	SE
		Europe	USA	Male	Female					
RS	*n*	27	54	47	34	81	73.20	0.8	6.7 (*n* = 77)	0.3
	%	13%	26.2%	22.8%	16.5%	39.3%				
F	*n*	11	14	16	9	25	73.80	2.1	6.1 (*n* = 25)	0.5
	%	5.3%	6.7%	7.7%	4.3%	12.1%				
P	*n*	13	7	14	6	20	75.40	1.7	11.8 (*n* = 20)	1.1
	%	6.3%	3.3%	6.7%	2.9%	9.7%				
PI	*n*	20	0	15	5	20	74.30	1.4	7.1 (*n* = 20)	0.5
	%	9.7%	0.0%	7.2%	2.4%	9.7%				
SL	*n*	6	4	5	5	10	74.50	2.2	7.6 (*n* = 9)	0.8
	%	2.9%	1.9%	2.4%	2.4%	4.8%				
CBS	*n*	3	6	5	4	9	73.56	1.8	5.7 (*n* = 9)	0.8
	%	1.4%	2.9%	2.4%	1.9%	4.3%				
OM	*n*	4	0	1	3	4	71.75	4.4	5.0 (*n* = 3)	1.5
	%	1.9%	0.0%	0.5%	1.4%	1.9%				
PGF	*n*	1	1	1	1	2	77.50	4.5	9.5 (*n* = 2)	5.5
	%	0.5%	0.5%	0.5%	0.5%	1.0%				
OTHER	*n*	27	8	17	18	35	75.26	1.3	7.3 (*n* = 25)	0.9
	%	13.1%	3.8%	8.2%	8.7%	16.9%				
Total	*n*	112	94	121	85	206	74.02	0.5	7.1 (*n* = 194)	0.2
	%	54.30%	45.6%	58.7%	41.2%	100.0%				

*RS* Richardson syndrome, *P* parkinsonism, *OM* predominant ocular motor dysfunction, *PI* predominant postural instability, *F* predominant frontal presentation, *CBS* predominant corticobasal syndrome, *SL* predominant speech and language disorder, *PGF* progressive gait freezing

### Immunohistochemistry and evaluation of tau pathologies

Formalin fixed, paraffin-embedded tissue blocks from the investigated cases were evaluated. Immunostaining for tau was performed with anti-tau PHF-1 (Ser396/Ser404, 1:2000; Gift of Peter Davies) and the AT8 antibody (Ser202/Thr205, 1:200, Invitrogen/Thermofischer, MN1020, Carlsbad, USA. For concomitant proteinopathies, we evaluated these cases for Aβ, TDP-43, and alpha-synuclein pathologies as well as for vascular lesions [33, 44].

We evaluated neuronal (tangles and diffuse cytoplasmic immunoreactivity and threads), astrocytic (tufted astrocytes and other morphologies pooled together), and oligodendroglial (coiled bodies together with threads in the white matter) tau pathologies using a semiquantitative score (none, mild, moderate, severe). The following anatomical regions were examined: The middle frontal gyrus, anterior cingulate, inferior parietal gyrus, superior and middle temporal gyrus, precentral gyrus, and occipital cortex (including the striate, para- and peristriate regions), hippocampus (pyramidal layers and dentate gyrus together), amygdala, the caudate-putamen, globus pallidus, thalamus and subthalamic nucleus (these are in one block), the midbrain tegmentum, substantia nigra, locus coeruleus, pontine base, tegmentum, and inferior olives of the medulla oblongata (together represented here as medulla oblongata for the conditional probability analysis), cerebellar white matter (threads and coiled bodies), and dentate nucleus (neuronal and rarely astroglial tau pathology). For the block containing the subthalamic nucleus and thalamus, neuronal tau pathology scores are provided for the subthalamic nucleus and astroglial and oligodendroglial for the thalamus.

### Conceptual approach and statistical analysis

Our approach contained three steps: (1) We used the mean of the semiquantitative scores total tau pathology in each examined region to generate heatmaps [15, 22]; (2) we described patterns and compared semiquantitative score of cellular tau pathologies (neuronal, astroglial, and oligodendroglial) in different anatomical regions; followed by (3) comparison of different anatomical regions and cellular pathologies to calculate conditional probabilities (see below), which region and which cellular pathology (i.e., neuronal, astroglial, or oligodendroglial) might precede another one. In addition, for total tau scores we performed binary logistic regression analysis to evaluate the effect of additional pathological variables and age.

We applied conditional probability analysis as reported recently for the evaluation of sequential stages of aging-related tau astrogliopathy (ARTAG) pathology [24]. Accordingly, we compared two regions in all combinations for discordance. This can mean that one region is affected (any score) while the other is not (negative). The cases included in this study showed clinical symptoms and, therefore, were not considered as presymptomatic where one could expect that many regions lack tau pathology. Therefore, we applied a modified strategy: for the dichotomic stratification score 1 represented if one region was affected moderately or severely, while score 0 was given if a region was not or only mildly affected. The null hypothesis was that region *A* being positive with moderate or severe scores of any or sum of tau pathology while region *B* being negative or showing mild score of any or sum of tau pathology and the region *A* being negative or showing mild score of any or sum of tau pathology, and region *B* being positive with moderate or severe scores of any or sum of tau pathology is equally likely; thus *A* and *B* region is affected (i.e., showing accumulation of tau pathology) at the same time (i.e., being in the same stage). Thus, this reflects whether one region accumulates tau pathology earlier than another one. McNemar’s test was used to assess the evidence against the null hypothesis. We generated a matrix for total tau and for different cellular tau types involving various anatomical regions where each cell in the matrix corresponds to a conditional probability that one region is involved before another one. Conditional probability was calculated using crosstab function of SPSS. SPSS Statistics Version 24 was used for statistical analysis. The conditional probability of region *A* to precede region *B* or vice versa was calculated as follows:

**Table:** 

Region A	Region B	
	Tau pathology present (moderate/severe)	Tau pathology not present or only mild
Tau pathology present (moderate/severe)	W	X
% of cases within region A	%	%
% of cases within region B	%	*Conditional probability A preceding B*
Tau pathology not present or only mild	Y	Z
% of cases within region A	*Conditional probability B preceding A*	%
% of cases within region B	%	%

If the conditional probability for one region was significantly higher than for the other region we interpreted that that this region was most likely to be affected before the other. We interpreted the results analogously to the measurement of observer agreement for categorical data [23, 25]. If the conditional probability was > 0.80 (and *p* value was < 0.01) we interpreted that that particular region is highly likely to precede another region. If the conditional probability was 0.61–0.80, then it was interpreted as substantial, 0.41–0.60 as moderate, and 0.21–0.4 as fair and < 0.21 as poor evidence that the involvement of one region precedes the involvement of the other one. If the conditional probability was high for both (> 0.80) and the *p* value was < 0.01, we interpreted the results based on the frequencies of involvement of the examined variable in regions.

Binary logistic regression models were additionally used to generate odds ratios (OR) and 95% confidence intervals (CI), where the presence of tau pathology (any type) in the examined anatomical regions were the dependent variables, and age, duration of illness and presence of AD-related pathology (i.e., amyloid plaques), Braak NFT stage, and argyrophilic grain disease (AGD)-related pathology were the independent variables. To generate dichotomic values for the binary logistic regression we used the sum of the dichotomic scores of cellular tau pathology scores dichotomized again with the same concept. This means that if the sum of the dichotomic scores (0 or 1) of each cellular tau pathology was 0 or 1 we used the value 0 and if the sum was 2 or 3 we used the value 1. In case the OR > 10 with a significant p value we interpreted this as high likelihood that two regions are affected together. In case OR < 1 we interpreted this as less likely that the two regions were affected together; eventually meaning they could be affected independently.

Heat maps and pathology patterns were generated for PSP-RS, PSP-F, PSP-P, PSP-S, PSP-CBS, and PSP-PI. For PSP-PGF (*n* = 2) and PSP-OM (*n* = 4) we generated heatmaps for an overview but did not include these cases in further analyses. For detailed statistical evaluation we pooled clinical PSP subtypes and additionally we evaluated PSP-RS separately as well. We did not include PSP cases where the clinical phenotype could not be clearly determined (“other”).

For conditional probability analysis we applied a significance level of 0.01 for McNemar’s test and 0.05 for logistic regression with multiple independent variables. We chose a lower significance level than the traditional 0.05 for McNemar’s test in order to reduce the likelihood of false-positive findings. However, for the sake of completeness, we separately indicated those comparisons where *p* value was < 0.05.

In addition, one-way ANOVA and Tukey post hoc tests were used to compare age at death and duration of illness in different clinical groups. Kruskal–Wallis with Mann–Whitney post hoc tests were used to compare the differences between duration of illness in six clinical groups and the scores of total tau load and tau cytopathologies in the clinical groups in different anatomical regions. For significant results we performed ordinal regression analysis with correction for presence of AD pathology, age, and duration of illness. Chi^2^ square test was used to compare the frequencies of sexes in clinical groups. For these examinations *p* < 0.05 was considered significant.

## Results

### Demographic summary of cases

Demographic data of 206 cases included in the study are summarized in Table 1. The most frequent form is PSP-RS (*n* = 81). Age at death was available for all cases, while duration of illness was lacking in a few cases due to unclear records on symptom onset. Age at death did not show significant differences between groups. Duration of illness was significantly longer in cases with PSP-P than those with PSP-RS (*p* = 0.001), PSP-F (*p* = 0.001), PSP-CBS (*p* = 0.002) or PSP-PI (*p* = 0.002). There was no difference in the distribution of sexes within clinical groups.

### Spatial features of cellular tau pathologies distinguish PSP subtypes

**Fig. 1:**
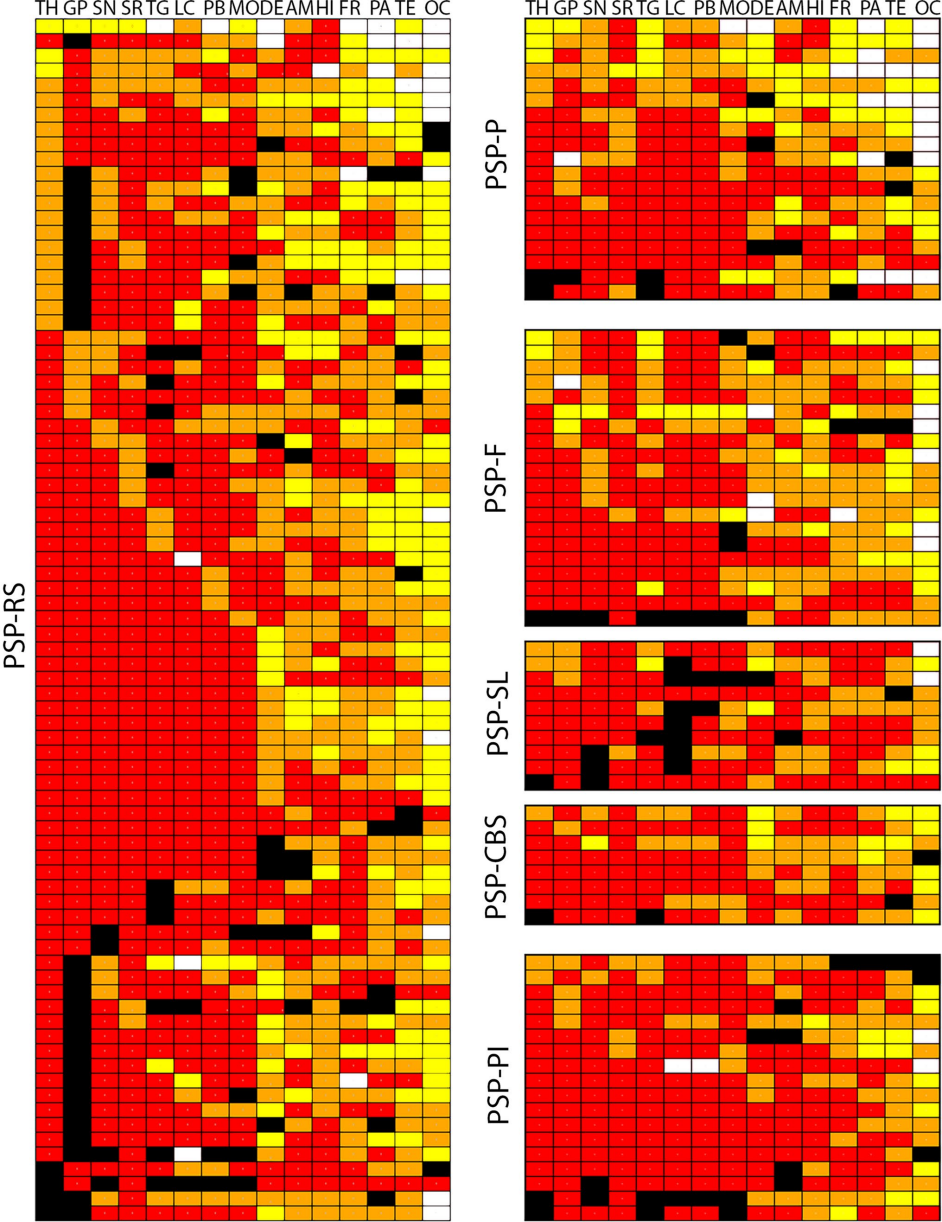
Distribution map of total tau semiquantitative scores (0–3) in PSP-Richardson syndrome (PSP-RS), PSP-frontal variant (PSP-F), PSP-Parkinsonism (PSP-P), PSP-postural instability (PSP-PI), PSP-speech-language variant (PSP-SL) and PSP-corticobasal syndrome (PSP-CBS). Black boxes indicate that that anatomical region was not examined. White box indicates score 0, yellow score 1, orange score 2, and red score 3 for semiquantitative scoring. Columns indicate anatomical regions, rows indicate individual patients. If more than six brain regions were not available the cases are not shown in this figure. *OC* Occipital, *TE* temporal, *PA* parietal, *FR* frontal, *MC* motor cortex, *AM* amygdala, *HI* hippocampus, *ST* striatum, *TH* thalamus and subthalamic nucleus, *GP* globus pallidus, *TG* midbrain tegmentum, *SN* substantia nigra, *LC* locus coeruleus, *PB* pontine base, *MO* medulla oblongata, *DE/CB* dentate nucleus and cerebellar white matter

**Fig. 2:**
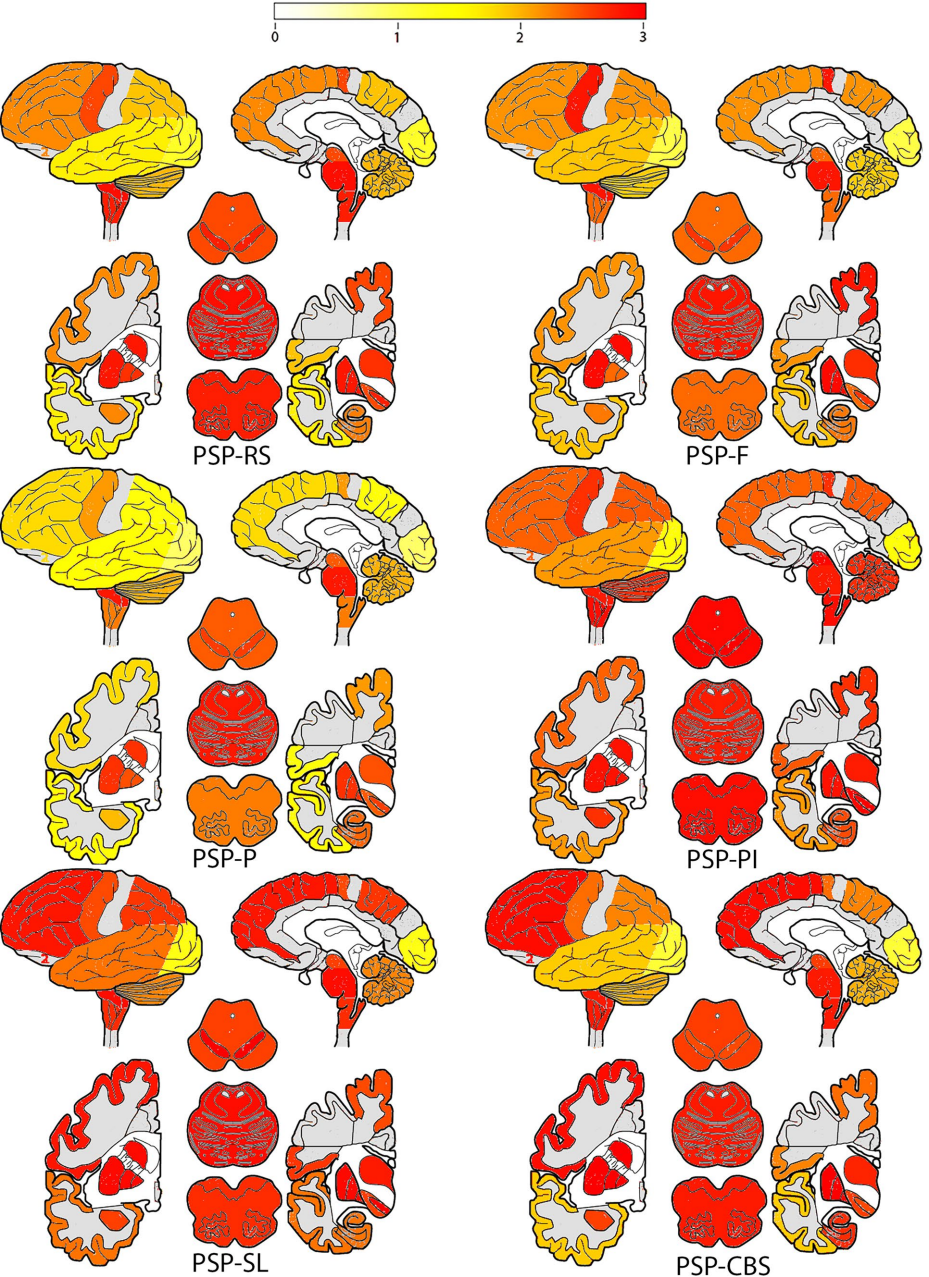
Heat mapping of total tau scores in PSP-Richardson syndrome (PSP-RS), PSP-frontal variant (PSP-F), PSP-Parkinsonism (PSP-P), PSP-postural instability (PSP-PI), PSP-speech-language variant (PSP-SL) and PSP-corticobasal syndrome (PSP-CBS). The severity of tau pathology ranges from white (none) through yellow and orange to red (severe). Gray colored cortical regions indicate that the region was not evaluated

Anatomical matrix of total tau scores of pooled clinical subtypes shows that tau pathology concentrates in subcortical and brainstem nuclei (Fig. 1). There were only a few cases with low tau scores in the thalamus/subthalamus but with higher scores in the striatum and globus pallidus. To highlight differences of anatomical vulnerability patterns between clinical subtypes, we generated heat maps of total tau scores (Fig. 2 and online supplemental file Fig. 1. for PSP-PGF and PSP-OM). Kruskal–Wallis test with Mann–Whitney post hoc test revealed significant differences between clinical subtypes in several brain regions (Table 2). Following a correction for presence of AD pathology, age, and duration illness, total tau load was higher in PSP-RS than in PSP-P (globus pallidus) and was less than in PSP-PI in neocortical and brainstem regions, furthermore less than in PSP-SL and PSP-CBS in neocortical regions. In PSP-PI total tau load was higher than in PSP-F and PSP-CBS in brainstem regions as well as that in PSP-P in brainstem regions. Furthermore, PSP-P showed less total tau accumulation in neocortical regions and selected subcortical and brainstem nuclei than PSP-CBS and PSP-SL. Finally, PSP-SL and PSP-CBS showed more total tau load in some neocortical regions than PSP-F (for details see Table 2).

**Table 2:** Differences between total tau scores of PSP-Richardson syndrome (RS), PSP-frontal variant (F), PSP-Parkinsonism (P), PSP-postural instability (PI), PSP-speech-language variant (SL), and PSP-corticobasal syndrome (CBS)

	STN/ TH	GP	SN	ST	TG	LC	PB	MO	DE/CB	AM	HI	MC	FR	PA	TE	OC
RS/F								** > **								
RS/P		** > **														
RS/PI			** < **		** < **			** > **	** < **	** < **				< *	** < **	
RS/SL													** < **	< *	** < **	
RS/CBS													** < **			
F/P												> *		> *		
PI/F					** > **			** > **	** > **							
F/SL													** < **	< *		
F/CBS													** < **			
PI/P			** > **		** > **			** > **	** > **	** > **		> *		> *		
P/SL		** < **	** < **										** < **	< *	** < **	
P/CBS		** < **											** < **			
PI/SL																
PI/CBS			** > **		** > **				** > **							
SL/CBS																

Greater-than signs indicate which clinical phenotype (in the order shown in the left column) shows significantly more tau pathology in a specific region after correction for AD pathology, duration of illness, and age. Greater-than signs with and asterisk show the significant results in Mann–Whitney test, which were not confirmed in the regression model

*OC* Occipital, *TE* temporal, *PA* parietal, *FR* frontal, *MC* motor cortex, *AM* amygdala, *HI* hippocampus, *ST* striatum, *STN/TH* subthalamic nucleus and thalamus, *GP* globus pallidus, *TG* midbrain tegmentum, *SN* substantia nigra, *LC* locus coeruleus, *PB* pontine base, *MO* medulla oblongata, *DE/CB* dentate nucleus and cerebellar white matter

**Fig. 3:**
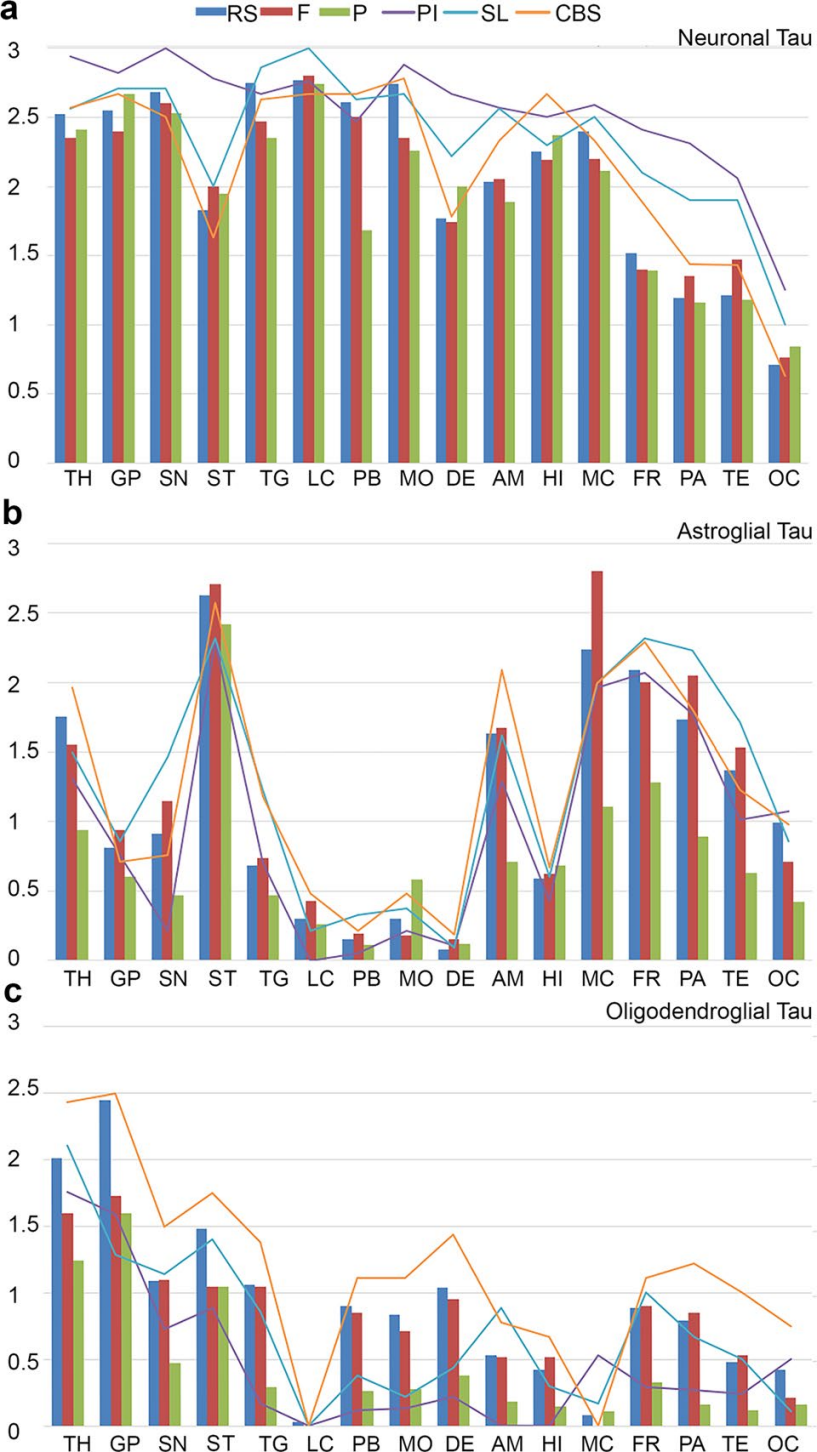
Graphic representation of semiquantitative scores of different cellular tau pathologies (**a** neuronal, **b** astroglial, **c** oligodendroglial) in PSP clinical subtypes. To avoid overcrowding of the graphs, three subtypes are shown in bars and three with lines. Significant (*p* < 0.05) results of Mann–Whitney test are shown for each comparison in D (blue box indicates neuronal, N, red box for astroglial, A, and green for oligodendroglial, O). *OC* Occipital cortex, *TE* temporal cortex, *PA* parietal cortex, *FR* frontal cortex, *MC* motor cortex, *AM* amygdala, *HI* hippocampus, *ST* striatum, *TH* thalamus *and* subthalamic nucleus, *GP* globus pallidus, *TG* midbrain tegmentum, *SN* substantia nigra, *LC* locus coeruleus, *PB* pontine base, *MO* medulla oblongata, *DE/CB* dentate nucleus and cerebellar white matter

Next, we compared the load of different cellular tau pathologies in clinical subtypes. Neuronal tau pathology affects mostly brainstem and subcortical nuclei but involvement of the amygdala and hippocampus is also considerable in all subtypes (Fig. 3a). Astroglial tau pathology clearly predominates in cortical areas and striatum and shows differences between clinical subtypes in all cortical areas, thalamus/subthalamus and substantia nigra (Fig. 3b). Furthermore, accumulation of oligodendroglial tau pathology is characteristic in subcortical nuclei and shows variability between subtypes in most of the cortical and subcortical and brainstem regions and cerebellum (Fig. 3c). Mann–Whitney test reveals significant differences between subtypes in several brain regions for each neuronal, astroglial, and oligodendroglial tau pathologies (Table 3). In logistic regression models, the duration of illness did not show any effect on these differences. Adding the presence of AD type pathology (i.e., presence of plaques), AGD, and age showed an effect on the results of differences of neuronal tau accumulation in the occipital and premotor cortex, amygdala and hippocampus but not in the frontal, parietal, and temporal cortices. While comparison of each clinical phenotype showed differences of at least one tau cytopathology in at least one region, major differences were noted in astroglial and oligodendroglial tau accumulation between PSP-RS and PSP-P, PSP-RS and PSP-PI, PSP-F and PSP-P, PSP-F and PSP-PI, PSP-PI and PSP-P, PSP-P and PSP-CBS, PSP-PI and PSP-SL and PSP-PI and PSP-CBS (Table 3). Neuronal tau accumulation was different mostly between PSP-RS and PSP-P, PSP-RS and PSP-PI, PSP-RS and PSP-SL, PSP-PI and PSP-F, PSP-P and PSP-PI and PSP-PI and PSP-CBS (Table 3). Neuronal loss correlated well with total tau load only in subcortical and brainstem regions and not in neocortical areas, likely due to the fact that cortical tau pathology was predominated by astroglial tau.

**Table 3:** Differences of cellular tau pathologies in PSP subtypes [PSP-Richardson syndrome (RS), PSP-frontal variant (F), PSP-Parkinsonism (P), PSP-postural instability (PI), PSP-speech-language variant (SL), and PSP-corticobasal syndrome (CBS)]

Clinical subtype	Tau/Cell	STN/TH	GP	SN	ST	TG	LC	PB	MO	DE/CB	AM	HI	MC	FR	PA	TE	OC
RS/F	Neuron								** > **								
	Astro																
	OligoD		** > **														
RS/P	Neuron					** > **		** > **	** > **								
	Astro	** > **		** > **							** > **		** > **	** > **	** > **	** > **	** > **
	OligoD	** > **	** > **	** > **		** > **		** > **	** > **	** > **				** > **	** > **	** > **	** > **
RS/PI	Neuron	** < **		** < **	** < **					** < **	** < **			** < **	** < **	** < **	** < **
	Astro			** > **			** > **										
	OligoD		** > **		** > **	** > **		** > **	** > **	** > **	** > **	** > **	** < **	** > **	** > **	** > **	
RS/SL	Neuron													** < **	** < **	** < **	
	Astro													** < **	** < **	** < **	
	OligoD		** > **						** > **	** > **							
RS/CBS	Neuron																
	Astroglia					** < **					** < **			** < **			
	OligoD																
F/P	Neuron														** > **		
	Astro			** > **							** > **		** > **		** > **	** > **	
	OligoD			** > **		** > **		** > **						** > **	** > **		
F/PI	Neuron	** < **			** < **									** < **	** < **	** < **	
	Astro			** < **			** < **										** < **
	OligoD					** > **		** > **	** > **	** > **	** > **	** > **		** > **	** > **		
F/SL	Neuron													** < **			
	Astro													** < **	** < **		
	OligoD																
F/CBS	Neuron																
	Astro										** < **						
	OligoD																** < **
P/PI	Neuron	** < **		** < **	** < **			** < **			** < **			** < **	** < **	** < **	
	Astro						** > **				** < **		** < **	** < **	** < **		** < **
	OligoD																
P/SL	Neuron							** < **			** < **						
	Astro			** < **		** < **					** < **		** < **	** < **	** < **	** < **	
	OligoD	** < **									** < **			** < **	** < **		
P/CBS	Neuron																
	Astro	** < **			** < **	** < **					** < **			** < **	** < **		
	OligoD	** < **		** < **		** < **		** < **	** < **	** < **				** < **	** < **	** < **	** < **
PI/SL	Neuron			** > **	** > **												
	Astro			** < **		** < **	** < **	** < **								** < **	
	OligoD										** < **			** < **			
PI/CBS	Neuron	** > **			** > **					** > **					** > **		
	Astro			** < **			** < **				** < **						
	OligoD					** < **		** < **	** < **	** < **	** < **	** < **		** < **	** < **	** < **	
SL/CBS	Neuron																
	Astro																
	OligoD		** < **						** < **	** < **							** < **

Greater-than signs indicate which clinical phenotype (in the order shown in the left column) shows significantly (Mann–Whitney test, *p* < 0.05) more cellular tau pathology (neuronal compared to neuronal, astroglial compared to astroglial and oligodendroglial compared to oligodendroglial) in a specific region. Note that differences of neuronal tau accumulation in the occipital and premotor cortex, amygdala, and hippocampus were lost after correction for age, duration of illness, presence of AD, and AGD pathology

*OC* Occipital, *TE* temporal, *PA* parietal, *FR* frontal, *MC* motor cortex, *AM* amygdala, *HI* hippocampus, *ST* striatum, *STN* subthalamic nucleus, *TH* thalamus, *GP* globus pallidus, *TG* midbrain tegmentum, *SN* substantia nigra, *LC* locus coeruleus, *PB* pontine base, *MO* medulla oblongata, *DE/CB* dentate nucleus and cerebellar white matter, *Astro* astroglia, *OligoD* oligodendroglia

The sum of the three different cellular tau pathologies is the highest in the striatum and the thalamus/subthalamic nucleus and the frontal, parietal, and motor cortices (exemplified by PSP-RS, PSP-P, PSP-F, PSP-PI, see online supplemental file, Figs. 2, 3, 4). This is due to the fact that brainstem nuclei accumulate less glial tau pathologies or only one type, such as oligodendroglial in the globus pallidus, pontine base, or cerebellum, and dentate nucleus.

**Fig. 4:**
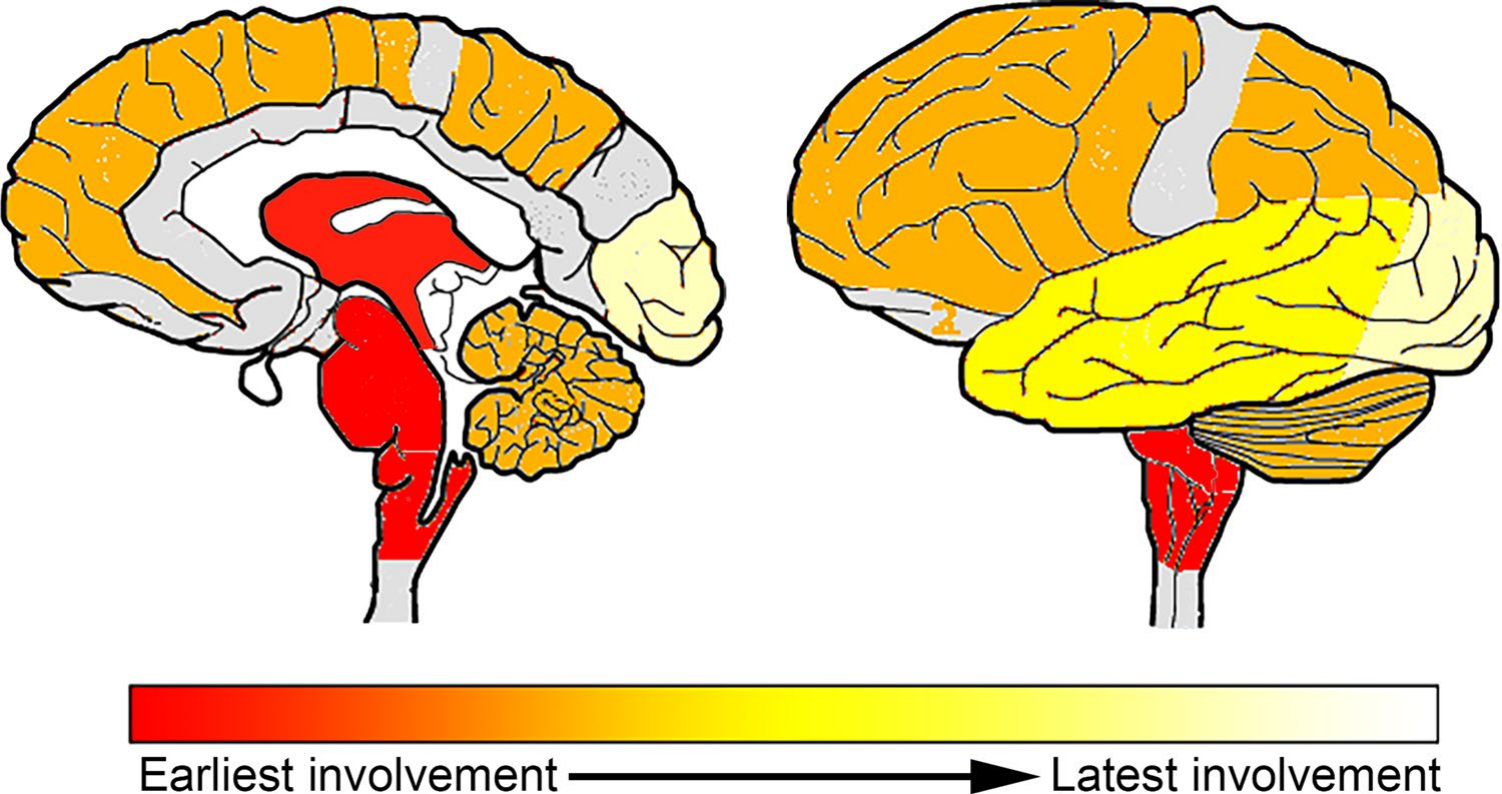
Heatmap showing the development of tau pathology based on conditional probability matrix of total tau pathology scores in pooled cases of different clinical subtypes. The dark red color indicates early and the yellow-white later involvement. Areas colored with gray were not included in the present study

Finally, we evaluated the combined effect of age, duration of illness, presence of AD-related pathology (plaques), Braak NFT stage and presence of AGD pathology on regional cellular tau pathologies. AGD pathology was found in 78 out 194 cases where diagnostically relevant regions were available for examination (40.2%, no difference between clinical phenotypes according to Chi^2^ test). Presence of AGD and Braak NFT stage showed significantly higher (*p* = 0.002 and 0.007, respectively) OR values for hippocampal neuronal tau pathology (OR 25.03, 95% CI 3.11–200.8, and 2.14, 95% CI 1.23–3.72, respectively). Neuronal and oligodendroglial tau pathology in the amygdala was significantly associated with the presence of AGD pathology (*p* = 0.0001, OR 10.16, 95% CI 2.8–35.6 and *p* = 0.006, OR 5.4 95% CI 1.6–18.6, respectively). Presence of astroglial tau pathology in the amygdala did not associate with presence of AGD (*p* = 0.4) or other variables in the model. Occipital cytopathologies were not influenced by these variables in this model.

In summary, total tau load is in general less in the cortex in PSP-P (and PSP-PGF in two cases) and more prominent in PSP-SL (Fig. 2). However, tau pathology in PSP shows clinical subtype and cell type-specific differences in its anatomical distribution. As general rules, the following were observed:

1. Subcortical and brainstem nuclei are most vulnerable for neuronal tau accumulation; however, the motor cortex is also involved;
2. Cortical areas and the striatum are characterized by predominance of astroglial tau pathology;
3. Oligodendroglial tau shows the most variability in clinical subtypes; and finally,
4. It is important to note that the amygdala and hippocampus consistently show tau pathology; however, neuronal tau pathology might indicate concomitant primary age-related tauopathy (PART) or AD or AGD.

### Conditional probability analysis for pooled PSP cases

We included in sum 165 pooled cases of PSP-RS, PSP-P, PSP-F, PSP-SL, PSP, PI, and PSP-CBS for this analysis. Our aim with this model was to evaluate whether the accumulation of tau pathology in a certain region precedes another, irrespective of the type of cellular tau pathology (online supplemental file, Figs. 5, 6). According to this, high or substantial evidence exists that involvement of subcortical regions precede cortical areas (first towards fronto-parietal cortices and then to temporal and finally occipital cortices) and dentate nucleus/cerebellar white matter (Fig. 4). This is reminiscent of the distribution map presented by Williams et al., in 2007, but the recognition of mild involvement of the occipital cortex found in our study expands beyond the regions shown there [48].

**Fig. 5:**
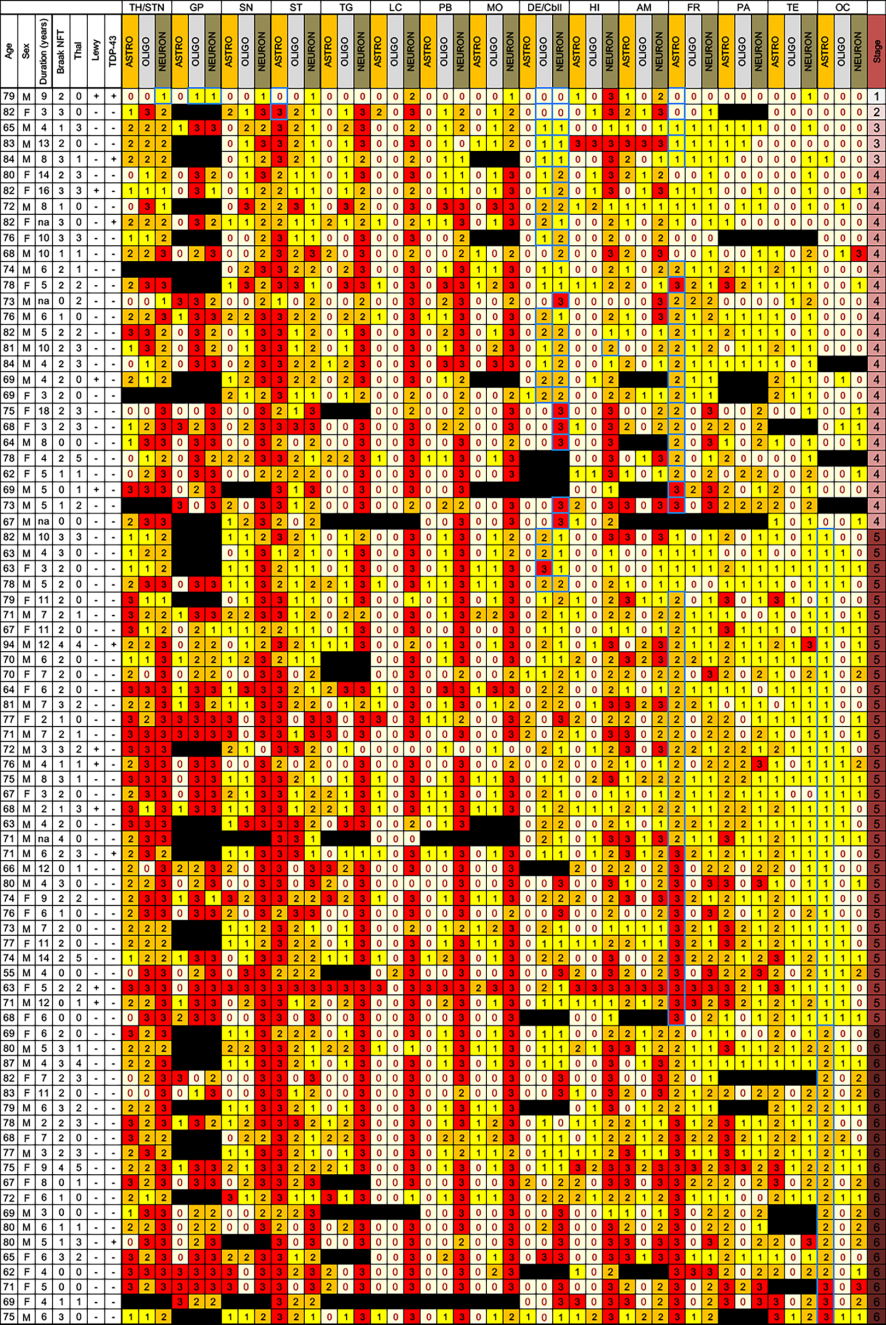
Distribution map of cellular tau pathology semiquantitative scores (0–3) in PSP-RS cases (*n* = 81). White box indicates score 0, yellow score 1, orange score 2, and red score 3 for semiquantitative scoring. Presence of Lewy-body pathology above Braak stage 2 or TDP-43 proteinopathy is indicated + , absence by −. Black boxes indicate that that anatomical region was not examined. On the right the proposed stages are indicated. Blue outlined boxes highlight the anatomical regions which were considered for the staging. *OC* Occipital, *TE* temporal, *PA* parietal, *FR* frontal, *MC* motor cortex, *AM* amygdala, *HI* hippocampus, *ST* striatum, *TH/STN* thalamus and subthalamic nucleus, *GP* globus pallidus, *TG* midbrain tegmentum, *SN* substantia nigra, *LC* locus coeruleus, *PB* pontine base, *MO* medulla oblongata, *DE/Cbll* dentate nucleus and cerebellar white matter

**Fig. 6:**
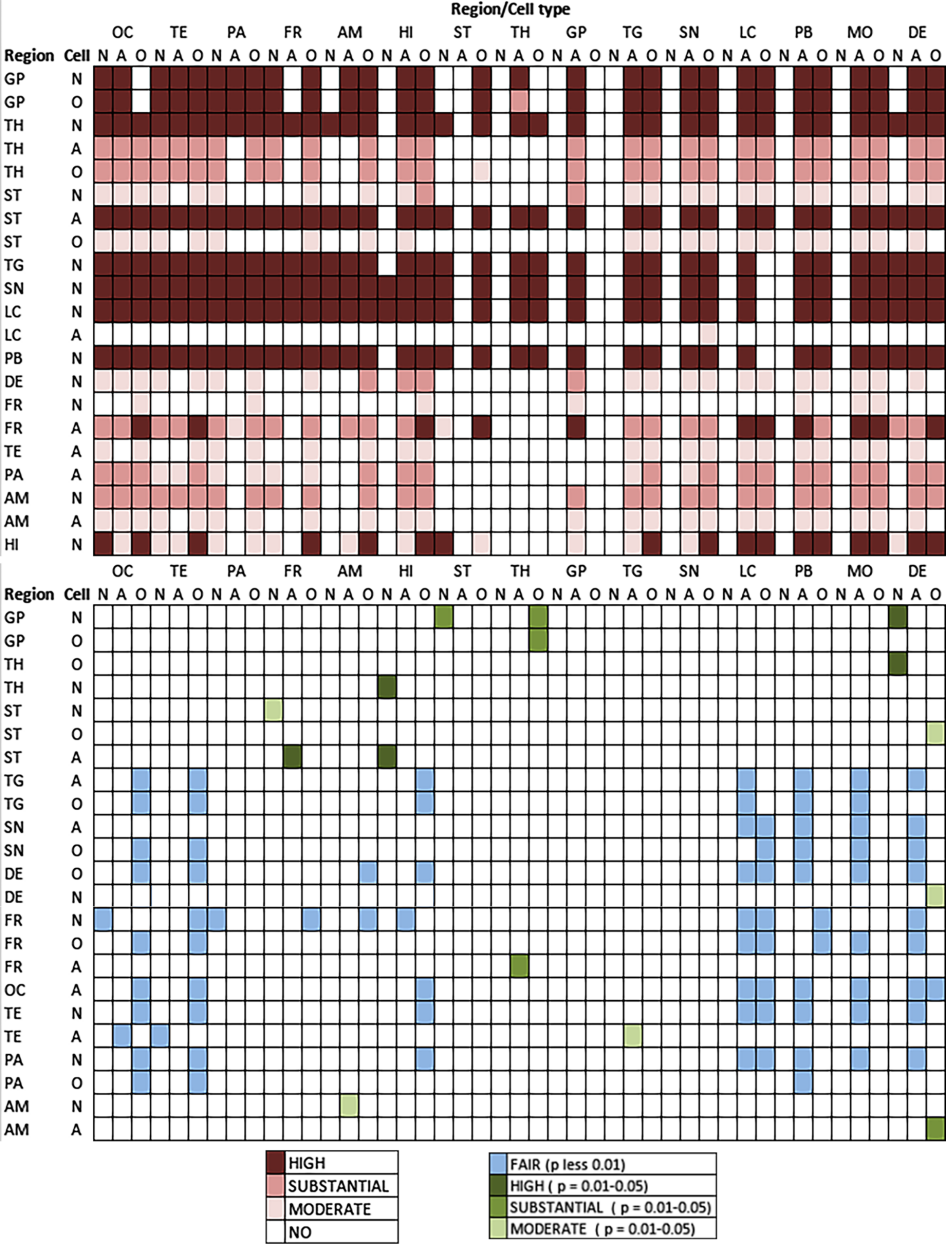
Summary of the conditional probability analyses (see online supplemental file). The upper image represents whether the anatomical regions listed in the left show high, substantial, or moderate probability with a p significance value below 0.01 to precede the involvement of the anatomical regions listed on the top. The lower image represents whether the anatomical regions listed in the left show high, substantial, or moderate probability with a p significance value below 0.05 or fair probability (with *p* < 0.01) to precede the involvement of the anatomical regions listed on the top.: *N* neuronal, *A* astroglial, *O* oligodendroglial, *OC* Occipital, *TE* temporal, *PA* parietal, *FR* frontal, *MC* motor cortex, *AM* amygdala, *HI* hippocampus, *ST* striatum, *TH* thalamus and subthalamic nucleus, *GP* globus pallidus, *TG* midbrain tegmentum, *SN* substantia nigra, *LC* locus coeruleus, *PB* pontine base, *MO* medulla oblongata, *DE/CB* dentate nucleus and cerebellar white matter

### Relation of tau pathological variables in different anatomical regions in Richardson syndrome

To predict early vulnerable cellular populations, we were next interested to discern which anatomical regions are affected when most others are spared any type of tau pathology. This analysis (Fig. 5) showed for PSP-RS (*n* = 81) that the subcortical nuclei (striatum, globus pallidus, subthalamic nucleus, and thalamus) and selected brainstem nuclei (substantia nigra, locus coeruleus, and medulla oblongata) show tau pathology in all cases. If the amount of tau pathology is low, then it was always neuronal rather than glial tau pathology in these regions. A single case with PSP-RS showed small amounts of neuronal tau pathology in the subthalamic nucleus, striatum, substantia nigra, and globus pallidus together with Lewy body and TDP-43 pathology. This observation supports the notion that these regions are early vulnerable regions for the development of the PSP-RS clinical phenotype. Following the development of a staging system (see below) we added the proposed stages to the cases (Fig. 5).

We provide the detailed table of conditional probabilities for PSP-RS cases together with the frequencies of low (0 and 1) and high (2 and 3) scores for each anatomical region and tau cytopathology in the online supplemental file Fig. 7, and here summarize the combined interpretation. Since the motor cortex was not examined in all cases of RS and the conditional probability results were similar as for the frontal cortex we show only the results of the frontal cortex.

**Fig. 7:**
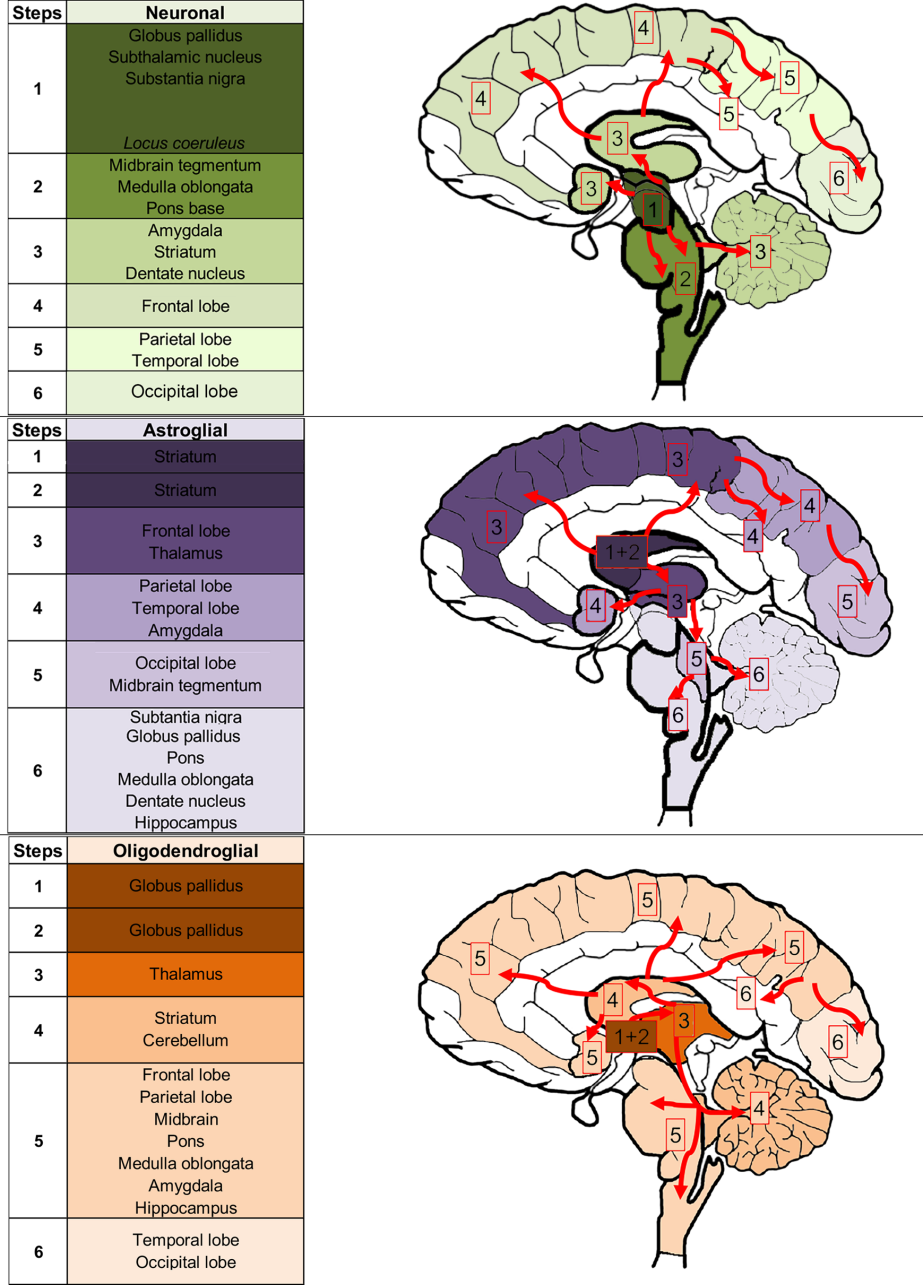
Sequences of PSP related tau pathology based on the conditional probability matrix and stratified for accumulation of neuronal, astroglial, and oligodendroglial tau pathologies. Note that frontal lobe includes frontal and motor cortices. Note that neuronal tau pathology is frequently seen in the hippocampus and locus coeruleus in stage 1; however, eventually this may be related to concomitant Alzheimer’s disease or primary age-related tauopathy (PART) pathogenesis. To indicate that locus coeruleus is frequently affected early, but alone might be associated with other disease conditions such as AD/PART, we used italic letters

Figure 6 demonstrates which cellular tau pathology in which anatomical region precedes one another with various degrees of likelihood. This shows that accumulation of neuronal tau pathology in the substantia nigra, midbrain tegmentum, locus coeruleus, pontine base, medulla oblongata, globus pallidus and subthalamic nucleus, and thalamus precedes any type of tau pathology in neocortical regions. Accumulation of neuronal tau in these regions precedes accumulation of neuronal tau in the dentate nucleus. Neuronal tau pathology accumulates later in the striatum but precedes neuronal tau accumulation in the parietal, temporal, and occipital cortices. The amygdala and hippocampus show accumulation of neuronal pathology earlier than neocortical neuronal tau. Amygdala is preceded by the accumulation of neuronal tau in the subthalamic nucleus and brainstem nuclei and hippocampus is preceded by the substantia nigra and locus coeruleus. In the cortex, accumulation of neuronal tau in the frontal cortex precedes that in the parietal and occipital cortices. Neuronal tau in the hippocampus and amygdala precedes accumulation of tau pathologies in cortical areas except for astroglial tau in the parietal and frontal cortices. However, early hippocampal involvement might be related to PART or AD pathology.

The striatum is different from other subcortical and brainstem nuclei, since here astroglial tau pathology accumulates more and earlier than neuronal tau pathology in the striatum and this occurs in parallel to the neuronal tau accumulation in other subcortical and brainstem nuclei. Astroglial tau pathology in the striatum precedes astroglial tau pathology in neocortical areas and thalamus. Importantly, in neocortical areas, except for the occipital cortex, significant values in conditional probability analyses suggest that astroglial tau pathology precedes the accumulation of neuronal or oligodendroglial tau pathology. Astroglial tau pathology accumulates later in the parietal, temporal, and occipital cortices, brainstem nuclei, hippocampus, and amygdala than in the frontal cortex. Astroglial tau in the occipital lobe and midbrain tegmentum precedes the locus coeruleus, medulla oblongata or dentate nucleus.

Finally, oligodendroglial tau pathology accumulates in the globus pallidus early and prominently and conditional probability analyses reveal a similar pattern as seen for the accumulation of neuronal tau. Oligodendroglial tau pathology in other regions does not seem to be accumulating early in the disease. Involvement of the subthalamic nucleus and thalamus and the striatum precedes neocortical areas and brainstem. Based on conditional probability analyses in the cortex the sequence seems to be frontal and parietal to temporal and occipital lobe.

Binary logistic regression was performed with multiple variables (i.e., age, duration, and presence of AD type pathology) to evaluate the odds ratios that two compared anatomical regions show accumulation of tau pathology simultaneously. This analysis confirms that neocortical areas are affected only if subcortical and brainstem nuclei are also affected (online supplemental file, Fig. 8). Importantly, it also shows that the involvement of the hippocampus and locus coeruleus is independent from the involvement of strategic subcortical and brainstem nuclei. On the other hand, tau pathology in the amygdala accumulates together with neocortical regions.

**Fig. 8:**
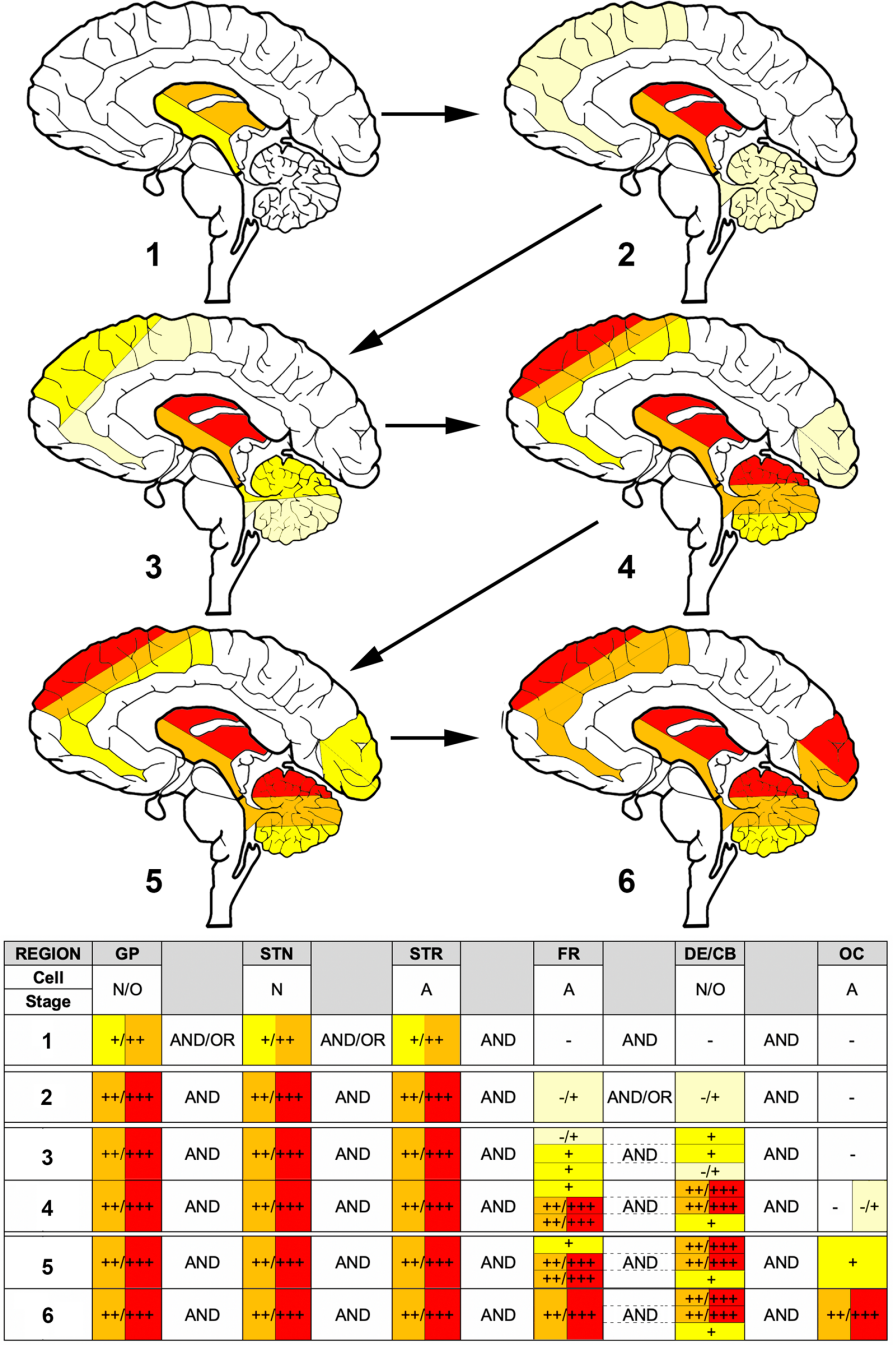
Proposed staging schema for the neuropathological practice. −/ +  Indicates single cell involvement; + indicates mild; +  + / +  +  + indicates moderate/severe involvement. *GP* globus pallidus, *STN* subthalamic nucleus, *STR* striatum, *FR* frontal, *DE/CB* dentate nucleus and cerebellar white matter, *OC* occipital. This can be applied to all clinical subtypes. The evaluator should focus on different cell types in different brain regions: in GP and DE/CB neuronal (N) or oligodendroglial (O); in the STN neuronal; in the STR and FR and OC cortices astroglial (A). The brain schema is a conceptual summary of the tabularized schema in the lower panel; thus the color coding of different brain regions reflect the variability in scores (or-or) required for a stage

### To summarize

Tau pathology in PSP-RS begins with neuronal tau accumulation in subcortical and brainstem nuclei along with early oligodendroglial involvement in the globus pallidus and astroglial involvement in the striatum. This is followed by astroglial tau accumulation in cortical areas, which precedes cortical neuronal and oligodendroglial tau accumulation, altogether following a fronto-parietal to temporal to occipital sequence.

## Discussion

The present study compared different clinical subtypes associated with PSP tau pathology and evaluated which cytopathologies in which anatomical regions precede others in PSP-RS. We show the following:

1. Common early vulnerability patterns characterize all PSP clinical subtypes jointly, i.e. affecting mainly the pallido-nigro-luysian axis;
2. Tau pathology propagates rostrally to neocortical regions and caudally to the cerebellum including the dentate nucleus;
3. Neuronal tau accumulation is the first step in the early affected pallido–nigro–luysian axis, but in some regions astroglial or oligodendroglial tau pathology precedes neuronal tau pathology;
4. Significant differences in tau burden, but particularly different tau cytopathologies, distinguish clinical subtypes.

### Common early vulnerability but distinct propagation patterns in PSP clinical subtypes

We applied different methods to confirm that the pallido–nigro–luysian axis is the early vulnerable region in PSP subtypes. This has been described in the original description of PSP [43] and emphasized in the diagnostic criteria [13] and in a model based on regional tau scores [48]. Importantly, neuronal tau pathology was observed in the locus coeruleus and hippocampus also; however, at least for PSP-RS, we showed that this was independent from the involvement of strategic subcortical and brainstem nuclei, and might represent or overlap with other pathogenic events such as AD or PART or, particularly for the hippocampus and amygdala, with the presence of AGD, which was frequently seen in our cohort (40.2%). On the other hand, observations on distinct subregional distribution of tau pathology in the hippocampus in PSP suggest that there is an AD/PART-independent pathogenic process of the involvement of the hippocampus [29].

Recently, several neuropathologic studies reported incidental or early stage PSP cases, supporting the importance of the pallido–nigro–luysian axis. Evidente et al. [8] reported five cases (6.9% of their cohort) with Gallyas-positive pathology fulfilling criteria of PSP but lacking clinical symptoms. They reported that the mean severity scores of Gallyas-positive PSP features were significantly lower in subjects with neuropathological incidental PSP than subjects with clinical PSP, with the subthalamic nucleus and putamen showing the greatest difference [8]. Kovacs et al. [21] reported five cases (2.1% in their cohort) with PSP pathology in an aging-study (not included in the present study), three of them without clinical symptoms but with tau pathology involving subcortical areas. Dugger et al. (2014) reported four cases (5% of their cohort, three of which were reported by Evidente et al. [8]). Three of their cases showed neuronal tau in the substantia nigra, two in the subthalamic nucleus and globus pallidus with variable involvement of cortical areas [7]. A single case showed tufted astrocytes in the cortex but no neuronal tau pathology in subcortical areas [7], which did not fulfil neuropathologic criteria of PSP. Nogami et al. [30] reported eight cases (2.5% in their consecutive autopsy cohort), which they termed preclinical PSP. All their cases showed neuronal tau pathology in the substantia nigra and either in the globus pallidus (6/8), subthalamic nucleus (4/8), putamen (5/8) or dentate nucleus (7/8) [30]. A single case contained tufted astrocytes in several regions [30]. Yoshida et al. [50] described 29 PSP cases (2.9%) in a forensic autopsy cohort, 13 of which showed low amount of pathology involving the globus pallidus, subthalamic nucleus, substantia nigra, and pontine nucleus. Many of them had clinical symptoms in spite of not being diagnosed as PSP [50]. Finally, a further case who deceased 2 months after the clinical onset showed neuronal degeneration only in the subthalamic nucleus and substantia nigra with more widespread tau pathology [38]. Importantly, the subthalamic nucleus is predominated by neuronal tau pathology, while other thalamic nuclei are affected by various cytopathologies showing differences between thalamic nuclei [12].

We theorize that from the common initiating sites in PSP clinical subtypes tau cytopathologies then propagate in different dynamics and patterns. Interestingly, intrinsic connectivity networks, anchored by the dorsal midbrain, whose nodes include the brainstem, basal ganglia, diencephalic, cerebellar and cortical regions have been recently established [10]. These outline a comprehensive architecture of node pairwise connections for this system and show that PSP–related connectivity breakdowns emphasize cortico-subcortical and cortico-brainstem interactions [10]. Several studies addressed the issue of distinct anatomical involvements as a basis for clinical variability, however, focusing on tau burden or neuronal tau pathology [17, 27, 36, 37, 39, 45]. The scoring system developed by Williams et al. considered coiled bodies and thread tau pathology as an important feature [48]. As a novel finding we report here that in addition to differences in overall total tau burden, neuronal, astroglial, and oligodendroglial tau pathologies involve clinical subtypes differently. In particular, neuronal tau differs the least, astroglial tau pathology differs mostly in neocortical areas, while oligodendroglial also in subcortical regions (see Fig. 3). Interestingly, PSP-P, which generally shows a slower disease progression than PSP-RS [41], differs mostly by the lower degree of glial involvement and particularly of cortical regions. This underpins the importance of glial tau pathology, which might reflect distinct propagation mechanisms of tau or differences in the response to neuronal degeneration [20]. The relevance of astroglial tau pathology has been discussed in distinguishing PSP and pallido–nigro–luysian degeneration [49]. Interestingly, early involvement of astrocytes is a feature of CBD [28] and has been also reported in brain regions not affected by neuronal tau in Pick’s disease [15, 24]. The discrepancy between neuronal and glial tau pathology in different PSP is intriguing also in the context of divergent patterns of transcriptional associations for neuronal and astroglial tau lesions [2]. Indeed, while neuronal tau pathology positively associated with a brain co-expression network enriched in synaptic and PSP candidate risk genes, astroglial tau pathology positively associated with a microglial gene-enriched immune network [2]. Finally, these observations carry a message for tau neuroimaging since the tracers should be able to detect glial tau also if these distribution patterns are targeted to be recognized. Importantly, PSP-PI showed several differences compared to other subtypes. As reported in other studies [32], we did not find difference in duration of illness for PSP-PI (except when compared to PSP-P). Early PSP-PI might resemble PSP-RS and accordingly does not show longer disease duration but associates with a significant neuropathological burden.

### Proposed sequential patterns of tau pathologies in Richardson syndrome

We recognize sequential distribution patterns that consider the accumulation of different cellular tau pathologies. Based on this and on reports on early stage, incidental, or preclinical PSP [7, 8, 30, 50], and as well as on reports on mapping of scores of tau pathologies [48], we propose a staging schema for PSP-RS. The conditional probability analysis used here focuses on the accumulation (dichotomized as no/mild versus moderate/severe) of any cellular tau pathology. Thus, we cannot exclude that single tau cytopathologies are not seen in a specific lower step of the sequential distribution in anatomical areas where accumulation is provided for a higher stage. Based on these concepts six sequential steps of tau accumulation can be recognized (Fig. 7). This is translated to six stages for practical neuropathological diagnosis:

*Step 1* of the sequence is characterized by the appearance of neuronal tau pathology in the globus pallidus, subthalamic nucleus, and substantia nigra. This vulnerability pattern has been already emphasized in the preliminary diagnostic criteria in 1994 [13] and by Williams et al. [48], who added that sparse tau pathology might be seen in the motor cortex as well. In this stage oligodendroglial coiled bodies can be observed in the globus pallidus and some degree of astroglial tau accumulation in astrocytes in the striatum. The locus coeruleus and hippocampus may also show neuronal tau pathology; however, this is most likely influenced by other pathogenic processes also and reflects concomitant AD or PART. Further studies should specify their exact contribution.

*Step 2* is characterized by accumulation of neuronal tau pathology in the midbrain tegmentum, medulla oblongata and pontine base, and astroglial tau pathology in the striatum. A few tau positive neurons may be seen in the striatum but this is less than the astroglial tau pathology in the striatum; thus the latter is a more consistent feature of this step. Oligodendroglial tau pathology further accumulates in the globus pallidus.

*Step 3* is characterized by the accumulation of neuronal tau pathology in the striatum, the dentate nucleus, and the amygdala, the latter influenced by concomitant AGD pathology. Neocortical areas (motor and frontal cortices, together representing frontal lobe) and subthalamic nucleus and thalamic nuclei show increased astroglial and oligodendroglial tau pathology.

*Step 4* is characterized by increased neuronal tau pathology in the frontal lobe; astroglial tau accumulates in the amygdala, parietal, and temporal lobe. Oligodendroglial tau accumulates in the striatum and cerebellar white matter.

*Step 5* is characterized by accumulation of neuronal tau pathology in the parietal and temporal lobes (involvement of this region is influenced also by concomitant AD/PART pathology). Astroglial tau increases in the occipital cortex and midbrain tegmentum, together with the accumulation of oligodendroglial tau pathology in frontal and parietal lobes and the brainstem (pons base, medulla oblongata, midbrain), as well as in the hippocampus.

*Step 6* is characterized by the rare situation that neuronal tau increases in the occipital cortex, as well as astroglial tau pathology accumulates, but never reaches severe degree, in the substantia nigra, globus pallidus, locus coeruleus, and medulla oblongata. Oligodendroglial coiled bodies may further accumulate in the occipital and temporal lobe.

For the practicing neuropathologists a simplified approach for the staging is summarized in Fig. 8. Single cellular tau immunoreactivity, defined arbitrarily as one tau immunoreactive cell in 20 high-power fields (× 40 objective) is not enough to define a stage. Although this study cannot exclude that tau pathology begins in the substantia nigra or the locus coeruleus, these regions might show single tau positive neurons in aging or concomitant AD/PART and, therefore, not included in the staging system. Apart from these aspects, a specific stage can be recognized if mild degree of tau pathology is seen.

To diagnose *stage 1* detection (mild/moderate degree) of neuronal tau pathology in the subthalamic nucleus and neuronal and/or oligodendroglial tau pathology in the globus pallidus and/or astroglial tau pathology in the striatum is required. *Stage 2* is characterized by prominent tau pathology in these regions with single cellular tau pathologies in the frontal cortex and/or dentate nucleus/cerebellum. *Stages 3 and 4* can be diagnosed if astroglial tau pathology accumulates in the frontal cortex and/or neuronal tau in the dentate nucleus and/or oligodendroglial tau pathology in the cerebellar white matter. Due to interindividual variability (i.e., rostral or caudal predominant progression), the dentate nucleus and cerebellar white matter might be discrepant from the frontal cortex and this can be indicated as rostral (i.e. cortical) or caudal (i.e. dentate/cerebellar) predominant. The difference between stages 3 and 4 is based on the amount of tau pathologies in these regions: either in the frontal cortex or in dentate nucleus/cerebellum or in both, tau pathology has to reach moderate or severe degree to allow recognition of stage 4. Single tau-positive astrocytes may be noticed in the occipital cortex. Stages 5 and 6 can be recognized if astroglial tau pathology accumulates (first mild then to moderate/severe degree) in the occipital cortex. This will less likely be seen in caudal predominant forms but will parallel increased amount of tau pathology in all other regions, however, with interindividual variability (Fig. 5).

The rationale to include occipital lobe in the staging is that when it is involved subcortical areas are so heavily affected that they cannot be evaluated to distinguish further stages. Although FDG-Positron Emission Tomography (PET) studies on PSP do not show significant hypometabolism in the occipital lobe [46], it must be noted that we also observe only relatively mild tau pathology. Furthermore, in this neuropathology staging system we focus on astroglial tau pathology, which might not associate with significant hypometabolism detectable by FDG-PET. Future studies using tau PET imaging are required to see whether this astroglial tau pathology is detectable in tau-imaging. Indeed, a study showing small amount of microscopically detectable tau pathology did not detect alterations in FDG-PET or tau-imaging in the occipital lobe [42]. Supporting our observations, current tau-imaging studies show that the subcortical areas are the primary affected regions in different clinical subtypes [40]; however, current tau-imaging may show off target binding and they have not been performed in end-stage PSP cases.

This staging requires five blocks to be stained for phospho-tau: (1) a block containing the globus pallidus and putamen, (2) the subthalamic nucleus, (3) frontal cortex, (4) cerebellum with dentate nucleus, and (5) occipital cortex. This staging system overlaps with the scoring strategy developed by Williams et al. [48], by emphasizing the central involvement of the pallido–nigro–luysian axis, basal ganglia, and dentate nucleus. However, contrasting that study, which focuses only on the accumulation of oligodendroglial coiled bodies and threads, we included astroglial tau pathology in our staging and define cortical areas also as important regions. Finally, we attempted to stage cases of various PSP clinical subtypes and acknowledged the practicality of this staging (online supplemental file Fig. 9). We noted that occasionally the parietal cortex might show more astroglial tau and can be additionally examined to diagnose stage 3. The overall aim of this staging is to be able to recognize early stages without or with only mild degree of clinical symptoms and these cases then can be evaluated as early or preclinical forms to understand earliest pathogenic events. Distinguishing frontal versus dentate/cerebellum predominant stages acknowledges different dynamics of propagation in various clinical subtypes.

### Limitations of the study

First, since we examined cases with obvious clinical symptoms and showing considerable tau pathology, our analysis is not able to predict precisely where exactly neuronal tau pathology begins in the brainstem or subcortical nuclei. Second, this model is based on the accumulation and not the presence of a single tau cytopathology; thus the difference between regions includes no/mild pathology compared to moderate/severe. Accordingly, the exact thresholds might need further validation. Third, the number of cases in less frequent clinical subtypes did not allow the application of a conditional probability matrix approach for each subtype. However, due to the common early vulnerability patterns as seen in the heatmaps, we evaluated the staging described for PSP-RS in other clinical subtypes and found that the cases can confidently be included in these stages. Fourth, we used a semiquantitative approach to evaluate tau pathology, which might not be able to distinguish differences within cases of the same scores. However, since image analysis methods are not yet able to distinguish tau cytopathologies and provide data only for total tau load, we used this strategy to assess cell-specific differences. Finally, tract-specific evaluation of white matter pathology was not specifically addressed since we were interested in distribution patterns in major anatomical regions, which are evaluated in the neuropathological practice, and according to this study, reflects well the progression in all clinical subtypes. However, this aspect can be evaluated in further studies to fine-tune subregional distribution patterns, which might better reflect interindividual variability.

## Conclusions

Our study supports the notion that the initiating site of neuronal degeneration and tau pathology seems to be similar in clinical subtypes, but the dynamics and propagation patterns distinguish them. While neuronal tau accumulation is central in the pathogenesis, astroglial, and oligodendroglial tau accumulation is important and may precede neuronal tau pathology in the striatum, cortical regions, globus pallidus, and cerebellar white matter (versus dentate nucleus). Finally, we propose a sequence of tau pathology in PSP-RS, which allows the recognition of a pattern of pathology and application of staging system. It seems that this might be applicable to various PSP subtypes; however, future studies should confirm this. This staging is recommended for the neuropathology practice using the blocks usually sampled in the diagnostic practice. This will allow standardized comparison of cases and recognition of early-stage cases in autopsy cohorts and in comparative studies with neuroimaging. However, future studies are needed to discover the basis of interindividual variability by evaluating further brain regions using other approaches, such as image analysis. Different clinical subtypes show different patterns of cellular tau pathologies; however, the regions included in the staging system parallel the accumulation of tau in strategic regions affected in all subtypes. Inclusion of the dentate nucleus/cerebellum and frontal cortex for staging can help to distinguish those subtypes, which involve more cortical region (“rostral predominant”) from those which predominate in brainstem and subcortical areas (“caudal predominant”). Since hypometabolism detected by FDG-PET correlates more with neuronal loss and not the accumulation of glial tau pathologies, difference in hypometabolism pattern might reflect better the clinical correlate for subtypes. Tau-based neuroimaging will help to clarify whether all tau cytopathologies are detectable and can help to distinguish clinical subtypes. Defining cell-specific stages of tau pathology helps to identify preclinical cases for the better understanding of early pathogenic events, has implications for understanding the clinical subtype-specific dynamics of disease-propagation, and informs tau-neuroimaging on distribution patterns.

## Electronic supplementary material

Below is the link to the electronic supplementary material.

Supplementary file1 (PDF 3081 kb)
