# Supplementary material for: Distribution patterns of tau pathology in progressive supranuclear palsy
Source: Acta Neuropathol. 2020 May 7;140(2):99–119. doi: 10.1007/s00401-020-02158-2 (PMC7360645; doi:10.1007/s00401-020-02158-2)
Supplement: Supplementary file 1 — Supplementary file1 (PDF 3081 kb) [file 401_2020_2158_MOESM1_ESM.pdf]

ONLINE SUPPLEMENTAL FILE

Distribution patterns of tau pathology in progressive supranuclear palsy

Suppl. Figure 1. Heat mapping of total tau scores in PSP with progressive gait freezing, (PSP-PGF), PSP with predominant ocular motor dysfunction (PSP-OM). The severity of tau pathology ranges from white (none) through yellow and orange to red (severe). Grey colored regions indicate that the region was not evaluated.

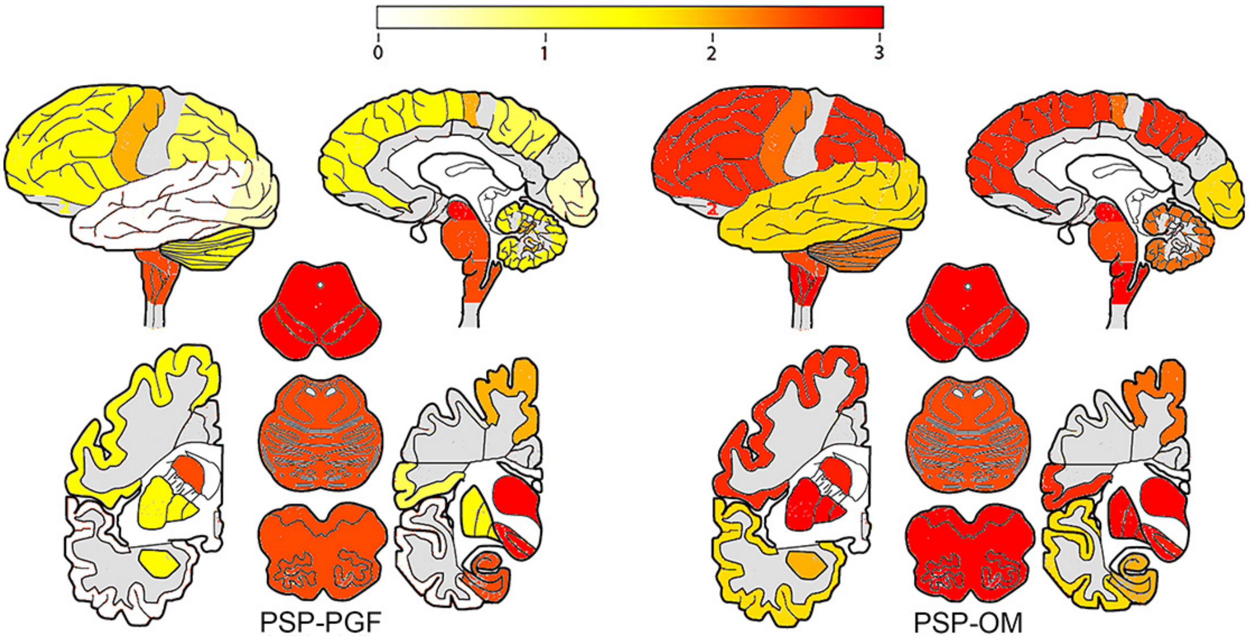

Suppl. Figure 2. Combined scores of neuronal, astroglial, and oligodendroglial tau pathologies in PSP-Richardson syndrome in different anatomical regions. The brown bars indicate the added semiquantitative scores of neuronal, astroglial, and oligodendroglial tau pathologies (minimum: 0 and maximum: 9). Black box means present, white box means not present. Blue box indicates high level of Alzheimer-related pathology. For the age one blue bar represent age less than 65 years, two bars age 66-75 and 3 bars age above 76 years. Presence of Alzheimer's diasease (AD, Lewy body or TDP-43 pathology is indicated by a black box. Blue box for AD indicates Braak stage above 3 with Thal phase above 2.

Abbreviations: OC: Occipital, TE: temporal, PA: parietal, FR: frontal, PM: premotor cortex, AM: amygdala, HI: hippocampus, ST: striatum, TH: thalamus and subthalamic nucleus together, GP: globus pallidus, TG: midbrain tegmentum, SN: substantia nigra, LC: locus coeruleus, PB: pontine base, MO: medulla oblongata, DE: dentate nucleus and cerebellar white matter.

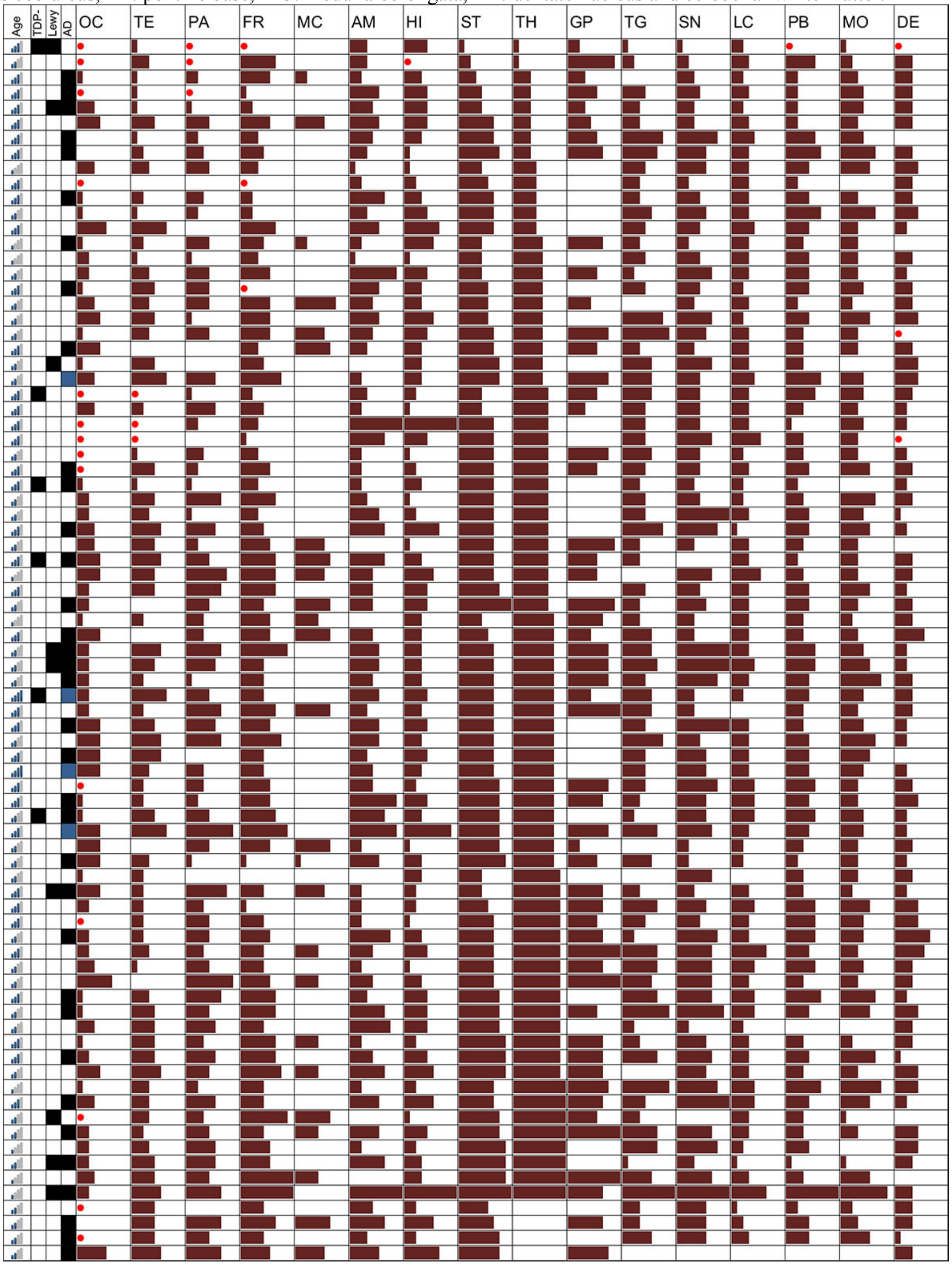

Suppl. Figure 3. Combined scores of neuronal, astroglial, and oligodendroglial tau pathologies in PSP-Parkinsonism (P), PSP-frontal variant (PSP-F), and PSP-postural instability (PI) syndrome in different anatomical regions. See legend for Suppl. 1/Figure 2 for coding of bars and regions.

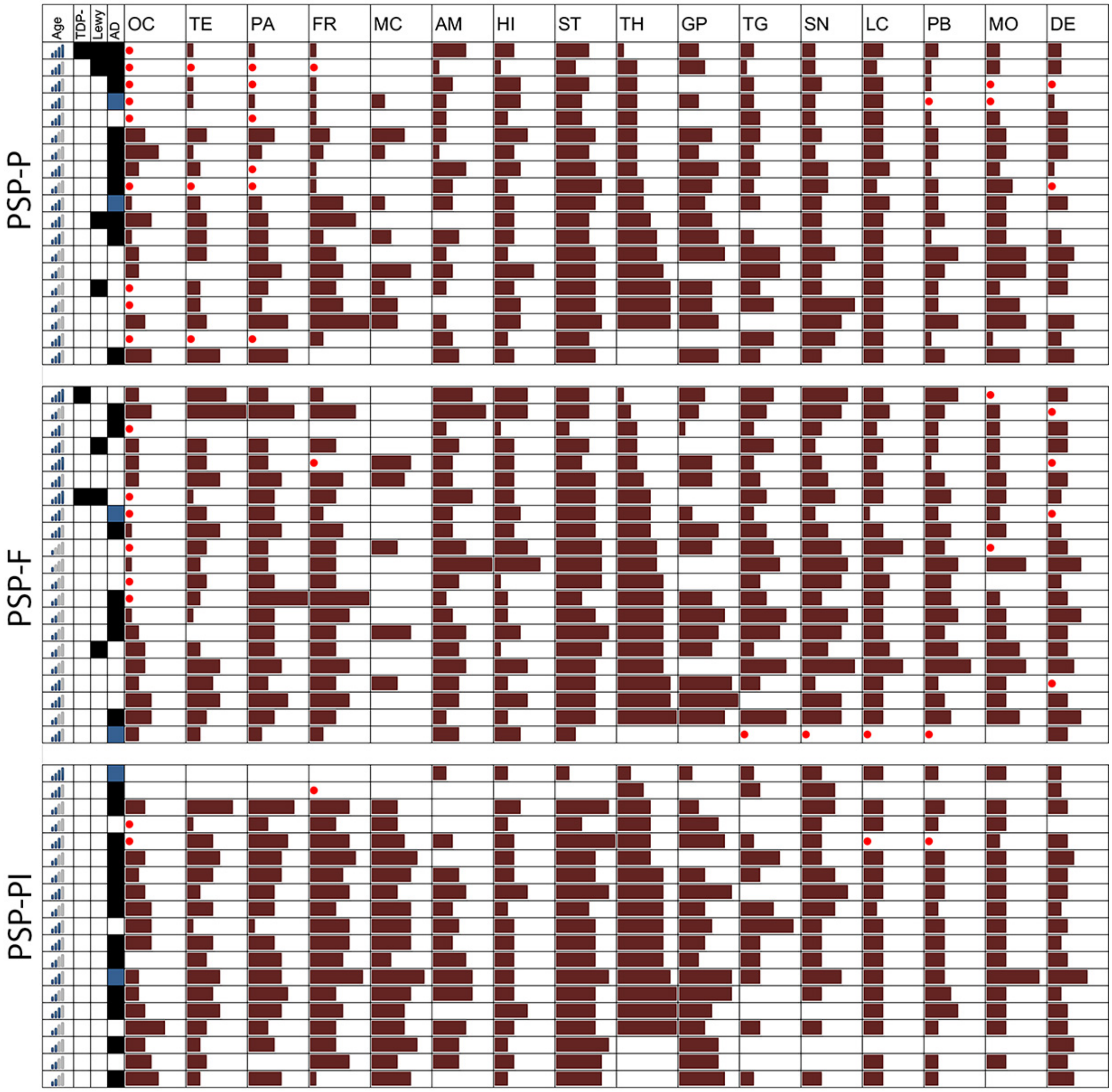

Suppl. Figure 4. Graphic representation the frequencies of cases with high (2 or 3) and low (0 or 1) scores in different anatomical regions in distinct clinical subtypes (PSP-RS, PSP-F, PSP-P, and PSP-PI).

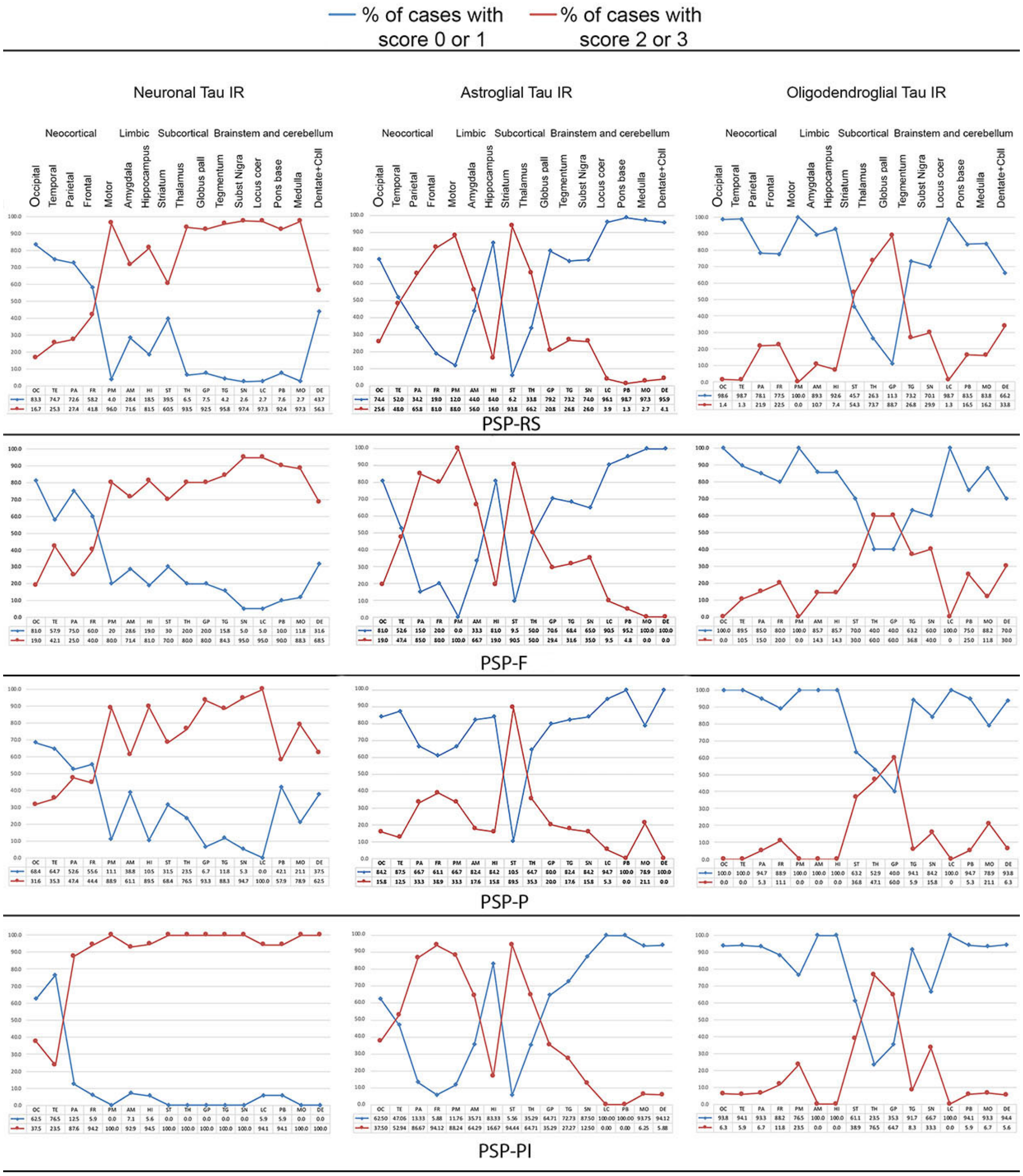

Suppl. Figure 5. Conditional probability matrix for all regions and total tau pathology in pooled cases of PSP-RS, PSP-P, PSP-F, PSP-SL, PSP, PI and PSP-CBS. Underlined and bold indicates $p < 0.01$, and bold indicates $p < 0.05$. If the conditional probability is high for both and significant we interpret the results based on the frequencies of involvement of the examined variable in regions; in this case the value associated to the more frequently invoved region is highlighted by red colour.

OC	OC	**0.16**	**0.23**	**0.14**	**0.14**	**0.15**	**0.15**	**0.5**	**0**	**0.21**	**0.33**	**0**	**0**	**0.12**	**0.31**
TE	**0.5**	TE	**0.25**	**0.26**	**0.1**	**0.38**	**0.6**	**0.6**	**0**	**0.57**	**0.33**	**0.75**	**1**	**0.5**	**0.48**
PA	**0.72**	**0.53**	PA	0.23	0.66	**0.63**	**0.55**	**0.22**	**0**	**0.74**	**0.33**	**1**	**1**	**0.37**	0.63
FR	**0.75**	**0.66**	0.44	FR	0.66	0.73	**0.36**	**0.4**	**0**	**0.3**	**0.33**	**0.75**	**0.66**	**0.12**	0.62
AM	**0.8**	**0.67**	0.79	0.75	AM	0.44	0.91	1	**0.5**	0.85	**1**	**1**	**1**	**1**	**0.91**
HI	**0.83**	**0.81**	**0.8**	0.82	0.52	HI	0.84	0.9	**0.5**	0.85	**1**	**1**	**1**	1	**0.88**
GP	**0.88**	**0.93**	**0.88**	**0.74**	0.95	0.9	GP	0.8	**0.5**	0.71	**0.33**	0.75	**0.66**	0.57	**0.88**
TH	**0.94**	**0.92**	**0.8**	**0.76**	1	0.95	0.85	TH	**0.5**	**0.42**	0.66	1	1	0.57	**0.87**
ST	**0.98**	**0.96**	**0.94**	**0.92**	**0.95**	**0.95**	**0.92**	**0.9**	ST	**0.85**	0.66	1	1	0.87	**0.94**
TG	**0.88**	**0.87**	**0.35**	**0.65**	0.9	0.9	0.71	**0**	**0**	TG	**0.33**	0.75	**0.66**	0.42	**0.75**
SN	**0.97**	**0.96**	**0.94**	**0.92**	**1**	**1**	**0.85**	0.87	0.5	**0.85**	SN	0.75	0.66	0.75	**0.91**
LC	**0.95**	**0.98**	**1**	**0.96**	**1**	**1**	0.91	1	1	0.91	0.66	LC	0	0.75	**0.97**
PB	**0.96**	**1**	**1**	**0.96**	**1**	**1**	**0.92**	1	1	**0.92**	0.66	0.25	PB	0.75	**0.97**
MO	**0.92**	**0.92**	**0.85**	**0.7**	**1**	1	0.72	0.62	0.5	0.69	0.33	0.5	0.33	MO	**0.79**
DE	**0.73**	**0.67**	0.63	0.51	**0.84**	**0.76**	**0.71**	**0.55**	**0**	**0.38**	**0**	**0.75**	**0.66**	**0.12**	DE

Suppl. Figure 6. Results of the binary logistic regression analyses corrected for age, duration of illness and presence of AD pathology (defined by presence of amyloid plaques). Orange box indicates odds ratios above 10 and significant.

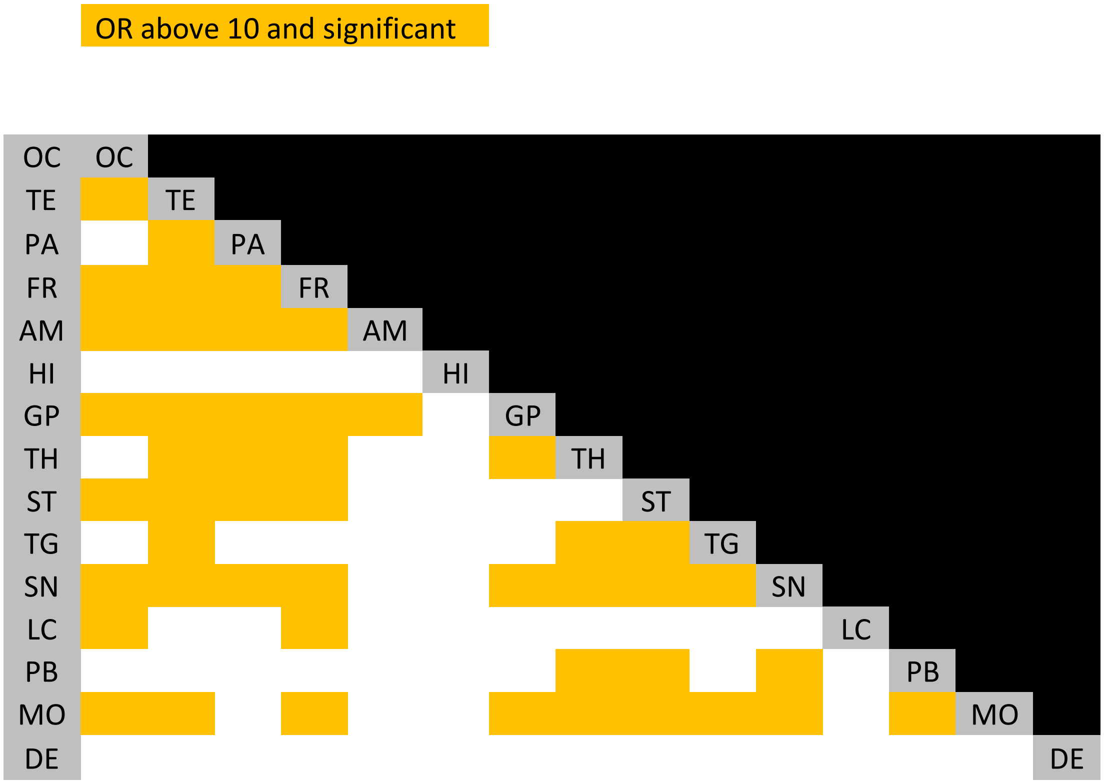

Suppl. Figure 7. Conditional probability matrix for all regions and tau cytopathologies examined. Underlined and bold indicates p < 0.01, and bold indicates p < 0.05. If the conditional probability is high for both and significant we interpret the results based on the frequencies of involvement of the examined variable in regions; in this case the value associated to the more frequently involved region is highlighted by red colour.

OCC-N	OCC-N	0.08	0.18	0.03	0.1	0.12	0.06	0.08	0.16	0.06	0.13	0.2	0.1	0.12	0.2	0.07	0.15	0.18	0.25	0.25	0.6	0.2	0.25	0.16	0.03	0	0.22	0	0.12	0.13	0	0.17	0.17	0	0.15	0.13	0.16	0.16	0.18	0	0.17	0.18	0	0.17	0.25
OCC-A	0.18	OCC-A	0.23	0.13	0.05	0.21	0.17	0.12	0.25	0.18	0	0.27	0.15	0.15	0.26	0.07	0.22	0.25	0	0.22	0.6	0	0.2	0.16	0.22	0	0.19	0	0.2	0.23	0	0.21	0.24	0	0.25	0.24	0	0.25	0.26	0	0.27	0.26	0.22	0.25	0.31
OCC-O	0.01	0	OCC-O	0.02	0	0	0.02	0	0	0.0	0	0	0	0	0.01	0	0.01	0.01	0	0	0	0	0	0	0.03	0	0	0	0	0	0	0.02	0	0	0.01	0.01	0	0.01	0	0	0.01	0	0.03	0.01	0.02
TEM-N	0.17	0.16	0.27	TEM-N	0.1	0.25	0.11	0.12	0.22	0.09	0.07	0.23	0.05	0.23	0.24	0.14	0.21	0.24	0	0.3	0.5	0.2	0.31	0.21	0.12	0.4	0.18	0	0.22	0.27	0	0.23	0.26	0	0.26	0.23	0	0.25	0.23	0	0.25	0.29	0.16	0.23	0.31
TEM-A	0.47	0.37	0.49	0.37	TEM-A	0.47	0.42	0.16	0.4	0.36	0	0.41	0.3	0.26	0.45	0.35	0.41	0.47	0.25	0.35	0.5	0.2	0.31	0.36	0.38	0	0.36	0.66	0.47	0.47	0.5	0.4	0.44	0.5	0.48	0.47	0.16	0.45	0.47	0.5	0.47	0.45	0.45	0.45	0.47
TEM-O	0.01	0	0	0.01	0	TEM-O	0.01	0	0	0.0	0	0	0	0	0.01	0	0.01	0.01	0	0	0	0	0	0	0.03	0	0	0	0	0	0	0.01	0	0	0.01	0.01	0	0.01	0	0	0.01	0	0.03	0.01	0.02
PAR-N	0.17	0.2	0.3	0.11	0.18	0.25	PAR-N	0.12	0.24	0.0	0	0.29	0.15	0.2	0.27	0.06	0.23	0.27	0	0.31	0.8	0	0.5	0.22	0	0.4	0.33	0	0.17	0.28	0	0.23	0.31	0.5	0.25	0.23	0	0.27	0.27	0	0.29	0.3	0.03	0.23	0.36
PAR-A	0.62	0.6	0.66	0.57	0.43	0.63	0.58	PAR-A	0.57	0.55	0.15	0.58	0.65	0.51	0.66	0.6	0.61	0.66	0.5	0.51	0.6	0.2	0.45	0.58	0.55	0	0.63	0.66	0.57	0.6	0.5	0.56	0.64	1	0.62	0.63	0.16	0.64	0.63	0.5	0.66	0.66	0.69	0.64	0.68
PAR-O	0.2	0.22	0.22	0.19	0.13	0.2	0.18	0.04	PAR-O	0.17	0	0.12	0.3	0.17	0.21	0.2	0.2	0.19	0	0.14	0.2	0	0.09	0.05	0.14	0.2	0.15	0.33	0.22	0.21	0.5	0.21	0.2	0.5	0.22	0.21	0.16	0.21	0.19	0.5	0.21	0.17	0.31	0.23	0.25
FRO-N	0.34	0.35	0.42	0.25	0.26	0.38	0.23	0.28	0.41	FRO-N	0.0	0.39	0.20	0.33	0.39	0.33	0.37	0.41	0.0	0.47	0.66	0.25	0.50	0.31	0.06	0.60	0.51	0.0	0.35	0.39	0.0	0.36	0.41	0.50	0.38	0.38	0.0	0.40	0.39	0.0	0.42	0.41	0.22	0.36	0.51
FRO-A	0.79	0.73	0.81	0.75	0.63	0.8	0.75	0.56	0.76	0.67	FRO-A	0.75	0.75	0.72	0.78	0.73	0.78	0.82	0.5	0.81	0.83	0.25	0.65	0.68	0.58	0.6	0.8	0.66	0.76	0.74	0.5	0.75	0.75	1	0.8	0.8	0.12	0.8	0.78	0.5	0.81	0.8	0.71	0.77	0.8
FRO-O	0.26	0.24	0.24	0.21	0.13	0.23	0.26	0.08	0.15	0.19	0	FRO-O	0.19	0.12	0.17	0.26	0.19	0.21	0.25	0.19	0	0.2	0.11	0.1	0.15	0.2	0.11	0.66	0.21	0.19	0.5	0.21	0.16	0.5	0.23	0.23	0.16	0.22	0.18	0.5	0.2	0.18	0.32	0.21	0.18
AMY-N	0.69	0.67	0.72	0.62	0.61	0.7	0.66	0.68	0.73	0.63	0.64	0.7	AMY-N	0.57	0.68	0.23	0.65	0.7	0.75	0.71	0.83	0.6	0.75	0.78	0.64	0.6	0.75	1	0.63	0.72	1	0.63	0.72	1	0.7	0.71	1	0.7	0.72	1	0.7	0.78	0.66	0.7	0.78
AMY-A	0.52	0.49	0.55	0.54	0.4	0.55	0.54	0.39	0.54	0.51	0.4	0.5	0.33	AMY-A	0.55	0.23	0.46	0.56	0.25	0.47	0.33	0.2	0.37	0.52	0.46	0	0.54	0.66	0.47	0.5	0.5	0.45	0.49	0.5	0.54	0.56	0.66	0.54	0.53	0.5	0.55	0.55	0.58	0.55	0.63
AMY-O	0.13	0.11	0.12	0.09	0.1	0.11	0.11	0.08	0.07	0.11	0.06	0.06	0	0.09	AMY-O	0	0.06	0.04	0	0.1	0	0	0.08	0.1	0.15	0	0.03	0	0.1	0.12	0	0.11	0.11	0	0.1	0.11	0.16	0.09	0.11	0	0.1	0.11	0.22	0.11	0.1
HIP-N	0.8	0.77	0.81	0.78	0.76	0.81	0.73	0.87	0.78	0.78	0.73	0.82	0.57	0.69	0.8	HIP-N	0.77	0.81	0.75	0.78	0.83	0.6	0.73	0.75	0.81	0.6	0.75	1	0.76	0.8	1	0.77	0.81	1	0.8	0.81	1	0.8	0.84	1	0.81	0.82	0.77	0.81	0.85
HIP-A	0.13	0.12	0.17	0.12	0.07	0.17	0.13	0.08	0.15	0.10	0.06	0.12	0	0	0.13	0	HIP-A	0.13	0.25	0.11	0.16	0	0.07	0.15	0.12	0	0.13	0	0.03	0.09	0	0.07	0.11	0	0.13	0.14	0.16	0.14	0.15	0	0.15	0.17	0.16	0.14	0.18
HIP-O	0.09	0.07	0.08	0.07	0.07	0.08	0.07	0.08	0.03	0.06	0.13	0.06	0.04	0.09	0.01	0.06	0.04	HIP-O	0	0.02	0	0.2	0.07	0.05	0.12	0	0.5	0	0.07	0.07	0	0.07	0.07	0	0.06	0.08	0.16	0.06	0.06	0	0.07	0.06	0.16	0.08	0.08
GP-N	0.91	0.89	0.91	0.87	0.89	0.91	0.87	0.88	0.9	0.81	0.71	0.92	0.9	0.87	0.9	0.9	0.93	0.92	GP-N	0.9	0.83	0.33	0.89	0.76	0.72	0.75	0.9	0.75	0.89	0.9	0.75	0.91	0.91	1	0.91	0.91	0.33	0.92	0.9	0	0.91	0.9	0.85	0.9	0.9
GP-A	0.18	0.16	0.22	0.12	0.1	0.16	0.09	0.1	0.18	0.04	0	0.2	0	0.08	0.2	0.18	0.15	0.21	0	GP-A	0.33	0.33	0.21	0.15	0	0.25	0.25	0	0.16	0.26	0	0.11	0.25	0	0.18	0.2	0	0.19	0.16	0	0.18	0.2	0	0.13	0.23
GP-O	0.94	0.94	0.88	0.93	0.92	0.91	0.96	0.88	0.9	0.90	0.85	0.85	0.9	0.82	0.86	0.9	0.88	0.88	0.75	0.9	GP-O	0.66	0.73	0.76	0.9	0.5	0.83	0	0.86	0.87	0	0.85	0.82	1	0.89	0.89	0.66	0.88	0.88	0	0.87	0.82	0.92	0.86	0.81
Thal-N	0.93	0.91	0.92	0.92	0.89	0.92	0.9	0.84	0.9	0.93	0.8	0.93	0.9	0.87	0.92	0.86	0.92	0.94	0.5	0.95	0.8	Thal-N	0.84	0.8	0.87	0.6	0.88	0.66	0.89	0.92	0.5	0.91	0.92	1	0.92	0.93	0.66	0.93	0.95	0.5	0.94	0.93	0.96	0.92	0.91
Thal-A	0.71	0.66	0.66	0.72	0.61	0.68	0.75	0.52	0.63	0.70	0.4	0.6	0.7	0.53	0.65	0.53	0.63	0.66	0.5	0.64	0	0.2	Thal-A	0.35	0.61	0	0.6	0.66	0.57	0.62	0.5	0.58	0.63	1	0.65	0.67	0.5	0.65	0.67	0.5	0.64	0.62	0.77	0.65	0.6
Thal-O	0.75	0.73	0.73	0.71	0.69	0.72	0.74	0.7	0.7	0.69	0.6	0.68	0.8	0.71	0.72	0.66	0.73	0.72	0.25	0.73	0.4	0.2	0.5	Thal-O	0.61	0.4	0.57	0.66	0.72	0.73	0.5	0.69	0.68	1	0.71	0.72	0.5	0.73	0.71	0.5	0.74	0.7	0.8	0.73	0.68
STR-N	0.53	0.58	0.6	0.51	0.51	0.59	0.49	0.52	0.59	0.37	0.13	0.56	0.47	0.48	0.59	0.6	0.58	0.62	0.25	0.71	0.93	0.2	0.53	0.4	STR-N	0.6	0.7	0.33	0.53	0.55	0.5	0.54	0.57	1	0.58	0.57	0.16	0.6	0.59	0.5	0.59	0.61	0.29	0.57	0.61
STR-A	0.92	0.91	0.93	0.94	0.87	0.93	0.94	0.8	0.93	0.95	0.86	0.93	0.9	0.84	0.92	0.86	0.92	0.93	0.75	0.92	0.66	0.6	0.8	0.85	0.93	STR-A	0.91	0.66	0.92	0.92	0.5	0.91	0.9	1	0.93	0.93	0.83	0.93	0.93	0.5	0.94	0.91	0.93	0.94	0.94
STR-O	0.56	0.5	0.54	0.51	0.46	0.55	0.58	0.52	0.5	0.63	0.53	0.48	0.61	0.54	0.52	0.4	0.52	0.5	0.25	0.45	0.16	0.2	0.46	0.25	0.65	0.4	STR-O	0.66	0.57	0.46	0.5	0.56	0.44	0.5	0.53	0.53	0.5	0.52	0.51	0.5	0.53	0.48	0.74	0.54	0.51
Tegm-N	0.95	0.94	0.95	0.94	0.97	0.95	0.94	0.95	0.95	0.93	0.93	0.98	1	0.98	0.94	1	0.95	0.95	0	0.97	0.75	0.8	0.95	0.94	0.93	0.75	0.96	Tegm-N	0.94	0.94	0	0.96	0.94	0.5	0.95	0.95	0.66	0.95	0.94	0	0.95	0.94	0.9	0.95	0.95
Tegm-A	0.26	0.23	0.25	0.27	0.26	0.26	0.26	0.2	0.28	0.23	0.2	0.25	0.15	0.19	0.27	0.14	0.19	0.26	0.25	0.28	0	0	0.04	0.23	0.2	0	0.31	0	Tegm-A	0.23	0	0.14	0.25	0.5	0.23	0.25	0	0.25	0.27	0	0.28	0.27	0.2	0.23	0.22
Tegm-O	0.26	0.25	0.24	0.31	0.26	0.26	0.34	0.25	0.26	0.27	0.13	0.23	0.35	0.22	0.28	0.28	0.24	0.26	0.25	0.28	0	0.2	0.13	0.23	0.23	0	0.12	0	0.23	Tegm-O	0	0.24	0.04	0	0.26	0.27	0	0.25	0.2	0	0.23	0.16	0.31	0.26	0.19
SN-N	0.96	0.96	0.97	0.96	0.97	0.97	0.96	0.96	0.98	0.95	0.93	0.98	1	0.97	0.96	1	0.96	0.97	0	0.97	0.83	0.8	0.96	0.95	0.96	0.8	0.97	0.33	0.96	0.96	SN-N	0.98	0.96	0.5	0.97	0.97	0.66	0.97	0.97	0	0.97	0.97	0.93	0.97	0.98
SN-A	0.27	0.21	0.25	0.25	0.2	0.27	0.23	0.12	0.24	0.22	0.06	0.25	0.09	0.12	0.26	0.07	0.18	0.25	0.25	0.2	0.16	0	0.08	0.15	0.16	0	0.28	0.33	0.14	0.24	0.5	SN-A	0.24	0.5	0.23	0.27	0.16	0.25	0.26	0.5	0.25	0.27	0.26	0.22	0.17
SN-O	0.3	0.28	0.26	0.33	0.3	0.3	0.35	0.24	0.28	0.31	0.13	0.23	0.33	0.21	0.29	0.28	0.26	0.29	0.25	0.32	0	0.2	0.24	0.2	0.25	0	0.14	0	0.24	0.02	0	0.28	SN-O	0	0.29	0.28	0	0.28	0.21	0	0.27	0.21	0.33	0.29	0.27
LC-N	0.96	0.96	0.96	0.96	0.97	0.97	0.98	1	0.98	0.97	1	0.98	1	0.96	0.96	1	0.96	0.97	1	0.97	1	1	1	1	1	1	0.97	0.66	0.98	0.95	0.5	0.98	0.96	LC-N	0.98	0.98	0.83	0.97	0.96	0.5	0.97	0.96	0.96	0.96	0.97
LC-A	0.04	0.05	0.04	0.05	0.05	0.04	0.03	0	0.02	0.02	0.06	0.03	0	0	0.03	0	0.01	0.03	0	0	0	0	0.04	0	0.03	0	0.03	0	0.02	0.04	0	0	0.4	0	LC-A	0.04	0	0.02	0.03	0	0.03	0.03	0.06	0.03	0.04
LC-O	0	0.02	0.01	0	0	0.01	0	0	0	0.0	0	0.01	0	0	0.01	0	0	0.01	0	0.02	0	0	0.04	0	0	0	0	0	0	0	0	0.02	0	0	0.01	LC-O	0	0.01	0.01	0	0.01	0.01	0	0.01	0.02
PB-N	0.92	0.89	0.92	0.89	0.87	0.91	0.88	0.8	0.9	0.86	0.66	0.91	1	0.93	0.92	1	0.92	0.93	0.5	0.9	0.83	0.6	0.88	0.85	0.83	0.8	0.91	0.33	0.88	0.88	0	0.91	0.88	0.5	0.91	0.92	PB-N	0.92	0.9	0	0.83	0.81	0.7	0.82	0.82
PB.A	0.01	0.02	0.01	0.01	0	0.01	0.02	0	0	0.0	0	0	0	0	0	0	0	0	0	0	0	0	0	0	0	0	0	0	0	0	0	0	0	0	0	0.01	0	PB-A	0	0	0	0	0.03	0.01	0
PB-O	0.17	0.15	0.11	0.14	0.17	0.15	0.19	0.16	0.14	0.13	0.06	0.11	0.23	0.15	0.18	0.33	0.16	0.15	0	0.14	0.16	0.4	0.19	0.1	0.12	0.2	0.13	0	0.17	0.09	0	0.17	0.07	0	0.16	0.17	0	0.15	PB-O	0.96	0.15	0.11	0.13	0.19	0.14
MO-N	0.96	0.96	0.96	0.96	0.97	0.97	0.95	0.95	0.98	0.95	0.92	0.98	1	0.96	0.96	1	0.96	0.97	0.75	0.97	0.83	0.8	0.96	0.94	0.96	0.8	0.97	0.33	0.95	0.95	0	0.98	0.96	0.5	0.97	0.97	0.51	0.97	0	MO-N	0.97	0.96	0.93	0.96	0.97
MO-A	0.03	0.03	0.03	0.03	0.02	0.03	0.04	0.04	0.02	0.02	0	0.01	0	0	0.01	0	0.01	0.14	0	0.02	0	0	0	0	0	0	0.03	0	0	0	0	0	0	0	0.01	0.03	0.06	0.01	0.01	0	MO-A	0	0.07	0.03	0
MO-O	0.17	0.15	0.12	0.21	0.16	0.16	0.2	0.4	0.13	0.14	0.93	0.12	0.35	0.16	0.16	0.21	0.17	0.01	0	0.15	0	0.2	0.12	0.05	0.17	0	0.08	0	0.17	0.08	0	0.17	0.07	0	0.16	0.17	0	0.15	0.09	0	0.14	MO-O	0.2	0.16	0.13
DE-N	0.44	0.53	0.56	0.46	0.54	0.53	0.41	0.59	0.6	0.41	0.4	0.61	0.47	0.55	0.6	0.46	0.55	0.6	0.5	0.64	0.83	0.8	0.69	0.66	0.24	0.5	0.75	0	0.48	0.55	0	0.57	0.57	0.5	0.55	0.53	0.37	0.57	0.53	0	0.56	0.57	DE-N	0.53	0.59
DE-A	0.03	0.04	0.04	0.03	0.05	0.04	0.02	0	0.05	0.0	0	0.03	0	0	0.04	0	0.01	0.04	0	0.02	0	0	0	0	0	0	0.06	0	0	0.04	0	0.02	0.04	0	0.03	0.04	0	0.04	0.05	0	0.04	0.05	0	DE-A	0.06
DE-O	0.4	0.4	0.33	0.42	0.37	0.35	0.44	0.39	0.36	0.47	0.4	0.31	0.5	0.43	0.33	0.46	0.35	0.33	0.25	0.33	0	0.2	0.21	0.22	0.36	0.25	0.29	0.33	0.33	0.29	0.5	0.27	0.3	0.5	0.33	0.36	0.04	0.32	0.3	0.5	0.3	0.29	0.38	0.34	DE-O

Suppl. Figure 8. Results of the binary logistic regression analyses for total tau in PSP-RS cases corrected for age, duration of illness and presence of AD pathology (defined by presence of amyloid plaques). Orange box indicates odds ratios above 10 and significant.

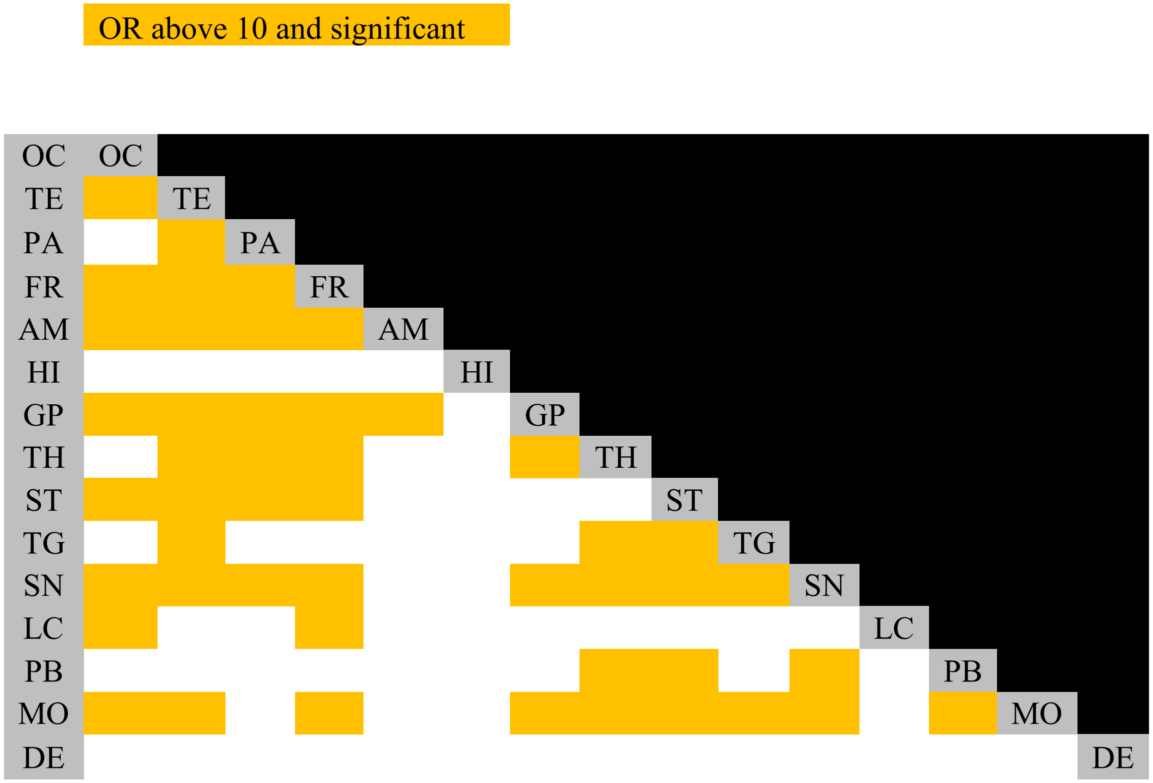

Suppl. Figure 9. Staging of PSP-related tau pathology developed by the evaluation of PSP-RS cases (see manuscript) in clinical subtypes PSP-P, PSP-F, PSP-SL, PSP-CBS, and PSP-PI. Gray colored columns indicate the strategic regions for staging. Red colored boxes indicate where discrepancy was observed and where the parietal cortex fits better to the stage then frontal cortex.

			TH/STN			GP			SN			SR			TG			LC			PB			MO			DE/CbII			AM			HI			FR			PA			TE			OC			
Age	Sex	FINAL CLINICAL	ASTRO	OLIGO	NEURON	ASTRO	OLIGO	NEURON	ASTRO	OLIGO	NEURON	ASTRO	OLIGO	NEURON	ASTRO	OLIGO	NEURON	ASTRO	OLIGO	NEURON	ASTRO	OLIGO	NEURON	ASTRO	OLIGO	NEURON	ASTRO	OLIGO	NEURON	ASTRO	OLIGO	NEURON	ASTRO	OLIGO	NEURON	ASTRO	OLIGO	NEURON	ASTRO	OLIGO	NEURON	ASTRO	OLIGO	NEURON	ASTRO	OLIGO	NEURON	Stage
79	1	P	0	2	1	0	2	2	0	0	2	1	1	1	0	0	1	0	0	2	0	0	1	0	0	2	0	1	1	0	0	1	0	0	1	0	0	0	0	0	0	0	0	0	0	0	0	3
94	1	P	0	0	1	0	2	1	0	0	2	3	1	1	0	0	2	0	0	3	0	0	2	0	0	2	0	1	1	2	0	3	0	0	3	0	0	1	0	0	1	0	0	1	0	0	0	3
81	2	P	0	0	3	0	0	3	0	0	2	2	0	2	0	0	2	0	0	3	0	0	0	0	0	1	0	0	1	0	0	2	1	0	3	0	0	1	0	0	1	1	0	0	0	0	0	3
81	1	P	1	1	1				2	0	1	3	1	1	0	0	2	1	0	2	1	0	0	0	0	0	0	0	0	1	0	2	1	0	3	1	0	0	0	0	0	1	0	0	0	0	0	3
78	1	P	1	1	1	0	3	3	1	1	2	3	2	1	0	1	2	2	0	2	0	1	0	0	1	1	0	0	1	1	1	3	1	1	2	1	0	0	0	0	0	0	0	2	0	0	2	3
67	1	P	0	2	2	0	3	2	0	1	3	3	3	1	0	1	1	0	0	2	0	1	1	0	1	3	0			1	1	1	0	1	1	1	0	0	0	0	0	0	0	0	0	0	0	3
78	1	P							0	2	3	3	1	1	0	2	3	0	0	3	0	0	1	0	0	1	0	1	1	2	0	1	0	0	2	1	0	1	0	0	0	0	0	0	0	0	0	3
72	2	P	0	0	3	2	0	3	0	0	3	3	0	3	0	0	3	0	0	3	0	0	2	0	0	3	0	0	3	0	0	2	2	0	3	0	0	3	2	0	2	0	0	3	0	0	3	4
81	2	P	0	0	3				0	0	2	2	0	2	0	0	3	0	0	3	0	0	1	0	0	3	0	0	3	0	0	2	0	0	2	0	0	1	0	0	0				0	0	0	4
82	1	P	2	1	3	3	0	3	0	0	3	3	0	2	0	0	2	0	0	3	0	0	1	0	0	3	0	0	2	1	0	3	0	0	3	1	0	1	1	0	2	0	0	3	0	0	1	4
68	1	P	2	3	3	0	2	3	0	0	3	2	2	2	0	0	3	0	0	3	0	0	2	0	0	2	0	0	3	1	0	1	1	0	2	2	0	2	1	0	2	1	0	1	0	0	0	4
82	1	P	1	0	3	2	0	2	0	0	3	3	0	3	0	0	3	1	0	3	0	0	2	1	0	3	0	0	3	0	0	3	0	0	3	2	0	3	0	0	2	0	0	2	0	0	1	4
74	1	P	3	2	3	1	1	3	3	2	3	2	2	2	2	0	3	0	0	3	0	0	2	2	1	3				1	1	2	2	0	2	3	0	2	2	0	0	2	0	0	0	0	0	4
68	2	P	2	2	3				0	0	3	3	0	3	3	0	3	0	0	3	0	0	3	3	0	3	0	0	3	0	0	3	3	0	3	3	0	2	3	0	2				0	0	2	4
72	1	P	2	2	2	1	3	3	2	1	2	3	1	2	3	1	2	1	0	2	1	1	3	1	2	3	0	2	2	1	0	1	0	0	2	2	1	1	2	1	0	1	1	1	1	1	0	5
66	1	P	2	3	3	0	3	3	1	2	3	3	2	2				0	0	3	0	2	3	2	1	3	1	1	2	0	1	1	1	1	2	3	3	3	2	2	2	1	1	1	1	1	1	5
75	1	P	0	2	3	0	2	3	0	0	3	1	2	3				0	0	3	0	0	3	0	0	3							0	0	3	3	2	2	0	0	3	0	0	3	2	0	2	6
79	1	P				0	3	3	0	0	3	2	2	2	0	0	3	0	0	3	0	0	3	0	0	2	1	1	3	2	0	2	1	0	2				3	0	3	3	0	2	2	0	2	6
67	2	P	0	0	3	0	0	3	0	0	2	2	0	3	0	0	2	0	0	3	0	0	2	0	0	3	0	0	3	0	0	1	0	0	3	1	0	2	1	0	2	0	0	1	2	1	2	6
80	1	F	3	1	1	0	1	1	0	1	1	3	1	2	0	1	1	0	0	1	0	1	1	0	1	1	0	0	0	1	0	2	0	1	3	1	0	1	2	1	1	1	0	2	0	0	0	2
75	1	F	0	0	3	0	0	1	0	0	3	0	0	2	0	0	2	0	0	2	0	0	2	0	0	2	0	0	3	0	0	2	0	0	1										0	0	0	3
72	1	F	1	3	3				1	2	3	1	3	3	0	2	1	1	0	3	0	2	2				0	2	2	2	1	1	0	0	1	2	1	1	2	1	1	1	1	1	0	0	0	4
48	2	F	0	3	3				1	3	3	3	2	2	0	3	3	0	0	3	0	3	2	0	3	3	0	3	2	3	3	3	1	3	3	2	1	1	1	1	1	0	1	1	0	0	1	4
85	1	F	1	2	2				1	1	3	3	2	1	0	1	3	0	0	3	0	1	3	0	1	2	0	1	1	2	1	3	0	1	2	2	1	1	2	1	1	0	0	1	0	0	0	4
63	2	F	3	0	3	3	0	2	3	0	3	3	1	3	2	0	3	3	0	3	0	0	3				0	0	3	2	0	3	3	0	2	2	0	2	2	0	1	2	0	1	0	0	0	4
75	1	F	2	3	3	2	3	3	0	0	2	3	0	3	0	0	3	0	0	3	0	0	2	0	0	3	0	0	0	2	0	2	0	0	2	2	0	2	2	0	1	2	0	2	0	0	2	4
72	1	F	2	2	3	3	0	3	3	0	3	3	2	3	3	0	3	0	0	3	0	0	3	0	0	3	0	0	3	3	0	2	1	0	3	2	0	2	2	0	2				0	0	2	4
73	1	F	1	3	3	0	2	3	0	1	2	3	0	1	1	1	2	0	0	3	0	1	3	0	0	2	0	1	2	1	0	1	0	0	2	3	3	3	3	3	3	1	0	1	0	0	0	4
92	1	F	0	0	1	0	2	2	2	2	3	3	1	1	0	2	3	0	0	3	0	2	3				0	1	2	1	2	3	0	2	3	0	1	1	1	1	1	2	1	3	1	1	0	5
65	1	F	2	2	3	1	3	3	2	2	3	3	1	2	2	2	3	0	0	3	0	2	3	0	1	2	1	2	2	2	0	1	0	0	2	2	2	2	2	1	1	1	0	0	1	0	0	5
67	1	F	3	2	2				3	2	3	3	2	1	2	2	3	3	0	3	2	2	3	1	2	3	0	2	2	3	1	1	2	1	2	3	2	1	2	2	1	2	2	1	1	1	1	5
83	1	F	1	3	1	0	3	3	0	1	3	3	1	2	0	1	3	0	0	3	0	1	3	0	1	2	0	1	1	2	0	1	1	0	2	3	1	1	3	1	1	3	1	1	1	0	0	5
84	1	F	1	0	2	0			0	0	2	3	1	1	2	0	3	0	0	3	0	0	2	0	0	2	0	1	2	2	0	2	1	0	2	3	0	1	2	0	1	2	0	1	1	0	1	5
78	2	F	2	0	2	2	0	3	2	0	2	3	0	3	0	0	3	0	0	3	0	0	3	0	0	3	1	0	2	2	0	1	2	0	1	3	0	2	3	0	2	3	0	2	1	0	1	5
93	2	F	0	0	3	1	1	3	0	0	3	2	0	2	0	0	2	0	0	2	0	0	1	0	1	1	0	0	0	0	0	3	0	0	3	0	0	0	2	0	1	1	0	2	1	0	1	5
68	1	F	3	2	2	0	3	2	1	1	2	3	2	2	0	1	1	1	0	3	1	1	3	1	1	3	0	1	2	2	0	2	0	0	1	2	1	1	2	1	1	1	0	1	2	0	1	6
84	2	F	3	2	3	3	3	3	1	2	3	3	0	3				0	0	3	0	0	3	0	0	3	0	0	3	2	0	2	2	0	3	3	1	2	3	0	3	3	0	2	2	0	2	6
68	1	F	0	1	1	0	2	1	1	2	3	3	1	1	0	2	2	1	0	3	1	0	2	0	0	2	0			3	2	3	0	2	3	3	3	1	3	3	1	3	3	3	2	1	1	6
72	2	F	3	3	3	1	3	3	2	2	2	3	1	2	2	2	3	0	0	3	0	1	3	1	1	3	1	2	2	0	0	2	0	0	2	2	1	1	2	1	1	1	1	1	2	1	1	6
72	1	SL	2	2	2				3	1	3	3	2	1	2	1	3	1	0	3	1	1	2	0	1	3	0	2	2	3	3	3	1	2	3	3	2	1	3	2	1	3	2	1	0	0	0	4
76	1	SL	2	2	3				1	3	2	3	2	1	2	3	3		0											3	1	3	1	1	2	3	2	2	3	2	2	2	1	2	0	0	0	4
83	2	SL	2	3	3	0	0	3				2	0	2							1	1	2	2	0	2	0	1	3	2	0	2	0	0	2	3	0	3	3		3	2	0	2	0	0	1	4
70	1	SL	3	3	3	2	2	3				2	2	3							0	0	3	0	0	3	0	1	3	2	0	2	1	0	2	2	1	3	2	1	2	1	1	1	0	0	1	4
77	2	SL		2	3	2	3	3	2	2	3	3	2	2	2	0	3	0	0	3	1	0	3	2	0	3	2	1	2	0	0	2	0	0	2	2	0	2	1	0	1	1	0	2				4
83	1	SL	1	2	1	0	2	1	3	0	2	3	1	1	0	0	2	0	0		0	1	3	0	1	3	0	1	1	2	0	2	0	0	2	3	1	1	3	1	1	2	1	1	1	1	0	5
59	1	SL	1	2	3	0	0	3	0	0	3	2	2	3	0	0	3				0	0	3	0	0	2	0	1	3	3	3	3	2	0	2	3	3	3	3	0	3	2	0	3	1	0	2	5
71	2	SL	3	3	2				3	2	3	3	2	1	2	2	3	0	0	2				0	0	2	0	1	1	2	1	3	0	0	2	2	1	1	2	0	1	2	0	1	2	0	0	6
78	2	SL				3	0	3	0	0	3	3	0	3	2	0	3				0	0	3	0	0	3	0	1	3				2	0	3	3	0	3	3	0	3	3	0	3	2	0	2	6
76	2	SL	0	0	3	0	2	3				3	1	3				0	0	3	0	0	2	0	0	3	0	1	2	0	0	3	0	0	3	3	0	2	3	0	2	2	0	3	3	0	3	6
80	2	CBS	2	2	2	1	3	3	1	1	2	3	2	1	1	1	2	0	0	3	0	2	3	0	2	3	1	2	2	2	3	3	0	3	3	3	2	2	3	2	1	2	1	1				4
73	1	CBS																0	0	3	0	0	3	0	0	3	0	0	2	3	0	2	2	0	3	2	0	3	2	2	2				0	0	0	4
72	1	CBS	2	2	2	1	3	2	1	2	2	3	2	1	0	2	3	0	0	2	0	2	3	1	2	3	0	1	1	2	0	1	0	0	3	3	2	1	2	2	1	0	1	1	1	1	0	5
69	2	CBS				0	3	2	1	1	3	3	2	2	1	1	3	1	0	3		1	3	1	1	2	0	1	1	2	1	2	1	1	3	3	1	2	2	1	1	2	1	2	1	1	1	5
71	2	CBS	3	3	3	0	3	3	2	3	3	3	2	1	2	3	3	0	0	3	0	3	3	1	1	3	0	3	1	3	1	3	1	1	2	3	1	1	2	1	1	2	1	1	1	1	0	5
82	1	CBS	3	2	3				2	1	3	3	2	1	2	1	3	1	0	3	0	1	2	0	1	3	0	2	2	3	2	3	1	1	3	3	2	1	3	2	1	2	1	1	1	1	1	5
65	1	CBS	3	3	3	0	0	3	0	3	3	3	0	3	2	2	3	0	0	2	0	0	3	0	0	2	2	3	3	3	0	3	0	0	3	3	0	3	2	0	3				1	0	2	5
72	1	CBS	3	2	3	3	3	3	0	0	3	3	1	3	3	0	3	3	0	3	2	0	3	2	2	3	0	0	3	2	0	2	2	0	2	3	0	3	2	0	2	2	2	3	1	1	1	5
78	2	CBS	0	3	2				0	1	1	3	3	1	0	1	1	0	0	2	0	1	1	0	1	3	0	1	1	2	0	2	0	0	2	1	2	1	1	1	1	0	0	1	2	1	0	6
90	1	PI	0	0	2	0	0	2	0	0	3	0	0	2		0	2	0	0	3	0	0	2	0	0	3	0	0	2	0	0	2	0	0	2													3
83	1	PI	1	0	3				0	2	3				1	0	2										0	0	2																			3
74	1	PI	0	2	3	0	3	3	0	0	3	2	0	2				0	0	3	0	0	2	0	0	3							0	0	2	2	0	2	1	1	1	0	0	1	0	0	0	4
84	1	PI	0	2	3	2	2	3	0	0	3	3	3	3	0	0	2	0	0	0	0	0	0	0	0	2	0	0	3	0	0	3	0	0	3	3	0	3	3	0	3	0	0	4	0	0	0	4
71	2	PI	2	2	3	0	0	3	0	0	3	3	0	3	0	0	3	0	0	3	0	0	3	0	0	3	0	0	3	2	0	3	0	0	3	2	0	3	2	0	3	1	0	2				4
70	1	PI	3	3	3	2	3	3	0		3	3	0	3				0	0	3	1	0	3	0	0	3	0	0	2	3	0	3	1	0	2	2	0	2	3	0	3	2	0	2	1	0	1	5
65	1	PI				0	3	3				3	2	3													2	0	2	2	0	2	0	0	2	2	0	2	2	0	2	1	0	1	1	1	1	5
77	1	PI	2	2	3	0	2	3	0	0	3	2	2	2	3	2	3	0	0	3	0	0	3	1	0	2	0	0	3	1	0	3	0	0	3	2	2	2			1	0	0	1	1	1	2	5
75	2	PI	2	2	3	2	3	3	2	2	3	3	2	3				0	0	3	0	0	3	0	0	3		0	3	2	0	3	2	0	3	3	0	3	2	0	2	0	0	2	1	0	2	5
76	1	PI	2	3	3	3	2	3	0	3	3	3	2	3	0	0	3	0	0	3	0	0	3	3	2	3	0	3	3	3	0	3	0	0	3	3	2	3	2	0	3	2	1	2	1	0	1	5
71	1	PI	2	0	3	0	0	3	0	2	3	3	2	3				0	0	3	0	0	3	0	0	3	0	0	3				2	0	2	3	0	3	2	2	3	2	2	3	1	1	1	5
72	1	PI	2	2	3	0	2	2	0	2	3	3	0	3	0	0	3	0	0	3	0	0	3	0	0	3	0	0	3	2	0	2	0	0	3	3	0	2	3	0	2	2	0	2	1	1	0	5
71	1	PI	0	2	3				0	0	3	3	0	3	3	0	3	0	0	3	0	0	3	0	0	3	0	1	3				0	0	3	3	1	3	2	1	2	2	0	3	1	1	1	5
75	2	PI	3	3	3	0	2	3				3	0	3				0	0	3	0	2	3	0	0	3	0	0	3				2	0	3	3	0	2	3	0	2	2	1	2	2	0	1	6
68	1	PI	2	2	3	2	0	2	2	0	3	3	0	2	2	0	3	0	0	2	0	0	2	0	0	3	0	0	2	2	0	1	1	0	1	2	0	2	2	0	2	2	0	2	2	0	2	6
78	2	PI	2	2	3	1	0	3	0	0	3	3	0	3	0	0	3	0	0	3	0	0	3	0		3	0	0	2	0	0	3	0	0	3	3	0	3	2	0	2	2	0	2	2	0	2	6
72	1	PI				2	2	3	0	0	3	2	2	3	0	0	2	0	0	3	0	0	2				1	0	3	0		3	0	0	2	0	0	1	2	0	3	0	0	2	2	0	3	6
66	1	PI				0	3	3				3	1	3				0	0	3	0	0	2	0	0	3	0	0	3	2	0	2	1	0	2	3	0	3				2	0	1	2	1	1	6
67	1	PI	3	3	3	1	0	3	0	0	3	3	0	3	0	0	3	0	0	3	0	0	2	0	0	3	0	0	3	2	0	3	0	0	3	2	0	2	0	0	3	0	0	3	2	2	2	6
